# Efficient generation of *Rosa26* knock-in mice using CRISPR/Cas9 in C57BL/6 zygotes

**DOI:** 10.1186/s12896-016-0234-4

**Published:** 2016-01-16

**Authors:** Van Trung Chu, Timm Weber, Robin Graf, Thomas Sommermann, Kerstin Petsch, Ulrike Sack, Pavel Volchkov, Klaus Rajewsky, Ralf Kühn

**Affiliations:** Max-Delbrück-Center for Molecular Medicine, 13125 Berlin, Germany; Present Address: Bayer Pharma AG Building S107, 13353 Berlin, Germany; Harvard University, Cambridge, MA 02138 USA; Berlin Institute of Health, Kapelle-Ufer 2, 10117 Berlin, Germany

**Keywords:** CRISPR, Cas9, Knock-in mice, Rosa26, Zygotes

## Abstract

**Background:**

The CRISPR/Cas9 system is increasingly used for gene inactivation in mouse zygotes, but homology-directed mutagenesis and use of inbred embryos are less established. In particular, *Rosa26* knock-in alleles for the insertion of transgenes in a genomic ‘safe harbor’ site, have not been produced. Here we applied CRISPR/Cas9 for the knock-in of 8–11 kb inserts into *Rosa26* of C57BL/6 zygotes.

**Results:**

We found that 10–20 % of live pups derived from microinjected zygotes were founder mutants, without apparent off-target effects, and up to 50 % knock-in embryos were recovered upon coinjection of Cas9 mRNA and protein. Using this approach, we established a new mouse line for the Cre/loxP-dependent expression of Cas9.

**Conclusions:**

Altogether, our protocols and resources support the fast and direct generation of new *Rosa26* knock-in alleles and of Cas9-mediated in vivo gene editing in the widely used C57BL/6 inbred strain.

**Electronic supplementary material:**

The online version of this article (doi:10.1186/s12896-016-0234-4) contains supplementary material, which is available to authorized users.

## Background

The *Rosa26* locus on chromosome 6 is frequently used for the integration of transgene constructs to achieve ubiquitous or conditional gene expression in mice. The *Rosa26* transcript is spliced into three exons and ubiquitously expressed in all cell types and developmental stages, but not translated to a protein [1]. The locus was first identified by the integration of the Rosaβ-geo (*r*everse *o*rientation *s*plice *a*cceptor βGal) gene trap vector in pool #26 of transduced embryonic stem (ES) cells [2]. This integration site, residing at the XbaI site within the first intron of Rosa26, has been used for ES-based gene targeting from its discovery on. A *Rosa26* targeting vector is extending 1 kb upstream and 4 kb downstream from the integration site within the first intron, flanking transgene inserts [3]. In the classical gene targeting procedure, targeted ES cell clones are injected into blastocysts to obtain germline chimeric mice and the transmission of targeted alleles to their offspring. This approach requires laborious handling of ES cell cultures and waiting times of 9–12 months until identification of positive F_1_ pups [4]. Nevertheless, the *Rosa26* locus is frequently targeted via ES cells for inserting single transgene copies in a standardized configuration into the mouse genome. The Mouse Genome Informatics database (MGI, www.informatics.jax.org) refers to 562 *Rosa26* knock-in mouse strains that have been generated for probing the effects of constitutively or conditionally expressed mutant proteins or for the imaging of reporter genes in vivo. *Rosa26* knock-in alleles are often configured such that coding regions are expressed under the control of the CAG hybrid promoter [5] or they are connected with splice acceptor elements to the endogenous *Rosa26* transcript [3]. Conditional gene expression is achieved by insertion of a loxP-flanked transcriptional stop element between the promoter and coding regions. In such a case, gene expression is induced by crossing the conditional knock-in line with transgenic mice expressing Cre recombinase in specific cell types [6].

Double-strand breaks (DSB) induced by engineered nucleases in mouse zygotes have emerged as powerful tool for the direct, single step production of targeted mutants, independent of ES cells. Proof of principle was provided with Zinc-finger nucleases and TALENs [7, 8], both of which have been largely displaced by the more versatile and efficient CRISPR/Cas9 gene editing system [9]. This system is composed of the generic Cas9 nuclease that is guided to specific target sites by short sgRNAs including 20 nucleotides complementary to the target sequence upstream of a PAM signal (NGG). Gene editing is achieved by endogenous DSB repair pathways, either imprecisely by non-homologous end joining (NHEJ) causing small deletions, or by homology-directed repair (HDR) using repair template vectors for the precise insertion of new sequences. In mouse zygotes, CRISPR/Cas9 has been efficiently used for generating small deletions and knockout mutations by the NHEJ repair pathway, reaching frequencies of 50 % in pups derived from RNA microinjections [10, 11], even in inbred backgrounds such as C57BL/6. In contrast, HDR events with co-injected targeting vectors occur rarely in zygotes. A limited number of studies reported the generation of knock-in alleles at frequencies of 5–15 % for a small number of genes [11, 12], not targeting *Rosa26* and using genetic hybrid embryos known for superior viability. Thus, an approach for the direct production of *Rosa26* knock-in alleles in C57BL/6 embryos is presently not established, despite this inbred background being a standard in biomedical research.

Here we applied CRISPR/Cas9 for the knock-in of conditional transgenes into *Rosa26* of C57BL/6 zygotes. Using modified Cas9 mRNA and sgRNA targeting the intronic XbaI site of *Rosa26*, compatible with common targeting vector homology regions, we achieved the knock-in of 8–11 kb inserts in 10–20 % of pups derived from microinjections of C57BL/6 embryos. This frequency increased to 50 % upon the combined microinjection of Cas9 mRNA and Cas9 protein, as tested in blastocyst assays. In addition to editing of the mouse germ line in zygotes, CRISPR/Cas9 offers a new perspective for modifying gene function in somatic tissues. To avoid the vector-mediated delivery of the large Cas9 transgene into primary cells, we generated *Rosa26* knock-in mice for the Cre/loxP-dependent expression of Cas9. Taken together, our protocols and resources support the fast and direct generation of new *Rosa26* knock-in alleles and of Cas9-mediated in vivo gene editing in the C57BL/6 background.

## Results

### Efficient DSBs induction at the *Rosa26* intronic XbaI site in C57BL/6 zygotes

**Fig. 1:**
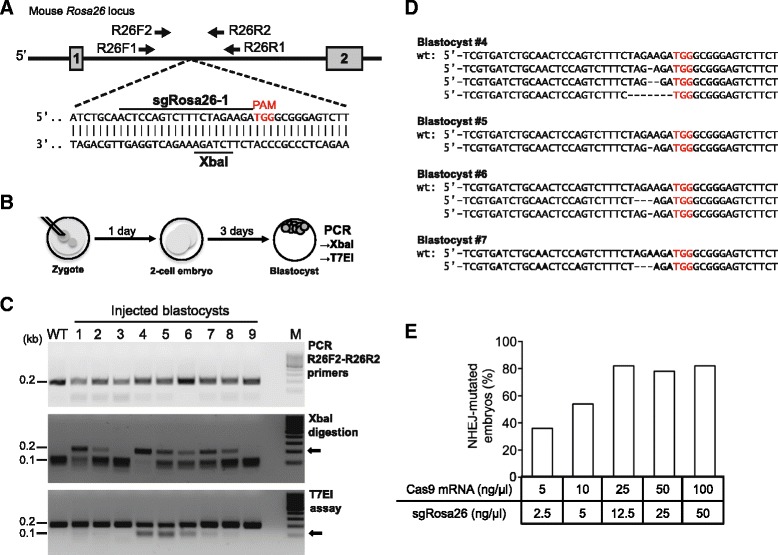
CRISPR/Cas9 induced DSBs at the *Rosa26* intronic XbaI site in mouse zygotes. **a**: Diagram of the mouse *Rosa26* locus. The sgRosa26-1 target sequence upstream of the protospacer adjacent motif (PAM) and the XbaI site within the first intron are indicated. The locations of primers used for nested PCR are shown (1. PCR: R26F1/R26R1, 2. PCR: R26F2/R26R2). **b**: In vitro blastocyst assay: zygotes microinjected with Cas9 mRNA and sgRosa26-1 RNA were cultured for 4 days to blastocysts. Genomic DNA was extracted from each blastocyst and used for PCR amplification of the target region and genotyping by XbaI or T7 endonuclease I (T7EI). **c**: Agarose gel electrophoresis of 0.2 kb PCR products amplified with the R26F2/R26R2 primer pair from blastocysts derived from microinjected zygotes (25 ng/μl sgRosa26-1 and 50 ng/μl Cas9 mRNA) (top). PCR products were either digested with XbaI (middle) or with T7EI (bottom). XbaI resistant 0.2 kb or T7EI sensitive 0.1 kb bands (arrows) indicate the presence of modified *Rosa26* alleles harboring sequence deletions. WT – wildtype control, M – size marker. **d**: Sequence comparison of cloned PCR products (from **c**) amplified from blastocysts #4 - #7 (from B). Deleted nucleotides are shown as dashes, the sgRosa26-1 PAM sequence is shown in red. **e**: Frequency of blastocysts showing NHEJ-based mutagenesis as indicated by the presence of XbaI resistant *Rosa26* PCR products, in relation to the concentrations of Cas9 and sgRosa26-1 RNAs used for the microinjection of zygotes

To achieve CRISPR/Cas9-mediated knock-in into *Rosa26,* we selected sgRNA target sequences spanning the XbaI site within the first intron, adapted to the homology regions of gene targeting vectors used for ES cells that cover sequences up- and downstream of this site [3]. As we have shown previously, sgRosa26-1 (Fig. 1a) exhibits high activity in mouse cells [13]. We therefore selected sgRosa26-1, together with a Cas9 mRNA that includes a plasmid coded polyadenine (polyA) tail (Cas9-162A) [14], for targeting in zygotes. The most effective concentrations of Cas9-162A and sgRosa26-1 RNAs were determined by microinjection of varying amounts of RNA into the pronuclei of C57BL/6 zygotes, followed by embryo culture to the blastocyst stage. Genomic DNA was extracted from each blastocyst and used for PCR amplification of the target region (Fig. 1b). PCR products were analyzed for NHEJ repair-associated deletions by digestion with XbaI or the T7 endonuclease I (T7EI). At the lowest concentrations of Cas9-162A (5 ng/μl) and sgRosa26-1 (2.5 ng/μl) RNAs, *Rosa26* alleles from 40 % of the embryos exhibited sequence deletions, as shown by the presence of XbaI resistant bands, whereas T7EI assays were less sensitive (Fig. 1c). Sequencing of cloned PCR products from four blastocysts confirmed the presence of small deletions at the expected cleavage site. Of note, individual deletion events could generate new XbaI sites, causing an underestimation of gene editing events by XbaI digestion (Fig. 1d). Upon RNA microinjection of Cas9-162A at 25 ng/μl and sgRosa26-1 at 12.5 ng/μl, 80 % of cultured embryos showed XbaI resistant PCR products, a percentage that was not further increased at higher concentrations (Fig. 1e, Additional file 1: Figure S1). XbaI resistant PCR products represented a minor fraction in most of the samples, indicating the preferential modification of the *Rosa26* allele in a heterozygous and/or mosaic pattern, although ~10 % of the embryos showed processing of both alleles. We reasoned that conditions leading to *Rosa26* deletions in the majority of embryos may also support knock-in events in at least a fraction of embryos, since HDR can occur in mammalian cells at ~10 % of nuclease induced DSBs [15].

### Knock-in of a conditional Cas9 transgene into *Rosa26* of C57BL/6 zygotes

**Fig. 2:**
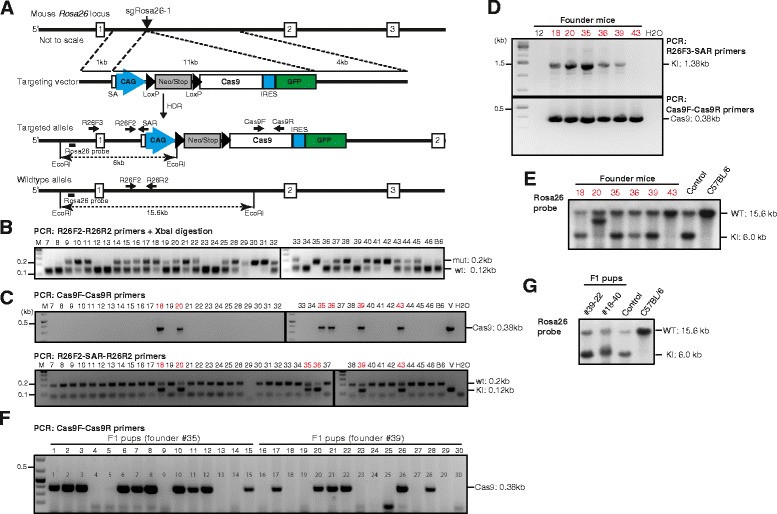
Knock-in of a conditional Cas9 transgene into *Rosa26* of C57BL/6 zygotes. **a**: Strategy for insertion of the CAG-loxPSTOPloxP-Cas9-IRES-EGFP cassette into the mouse *Rosa26* locus. sgRosa26-1 and Cas9 introduce a double-strand break between 1 kb and 4 kb fragments used as homology arms in the targeting vector. Homology-directed repair (HDR) leads to the insertion of the cassette into the genome. The locations of PCR primers, restriction sites and the *Rosa26* hybridisation probe in the targeted and wildtype alleles are indicated. **b**: Gel electrophoresis of XbaI digested *Rosa26* PCR products (R26F2/R2 primers) amplified from pups (#7-#46) derived from microinjections of targeting vector, sgRosa26-1 and Cas9 RNAs. 0.2 kb bands of XbaI resistant products (mut) indicate sequence deletions, wildtype products (wt) are reduced to 0.1 kb. M - size marker, B6 - C57BL/6 wildtype control. **c**: PCR detection of an internal segment of Cas9 in pups derived from microinjections using primers Cas9F/Cas9R (top). Bottom: three primer PCR for the simultaneous detection of the *Rosa26* target region (R26F2/R2 primers, 0.2 kb) and of vector sequences (R26F2-SAR, 0.12 kb), showing that all samples harbor at least one nonrecombined *Rosa26* allele. V – vector positive control, H_2_O – negative control. **d**: Cas9-positive mice (from **b**) were further tested for correct knock-in (KI) into *Rosa26* using a PCR reaction with a forward primer located outside of the 5′-homology region (R26F3) and a reverse primer located in transgene (SAR); the predicted band has a size of 1.38 kb (top). Bottom: DNA quality was controlled with a Cas9 internal PCR (Cas9F/R primers,0.38 kb). H_2_O – negative control. **e**: Southern blot analysis of EcoRI digested tail DNA from Cas9-positive mice (from **b**) using an external *Rosa26*-specific hybridization. Knock-in alleles are predicted to show a 6 kb band. Control – DNA from a *Rosa26* knock-in mouse generated from ES cells, C57BL/6 – wildtype control. **f**: Genotyping PCR of 15 F1 pups derived from founder mutants #35 or #39 using the Cas9 internal primer pair Cas9F/R. **g**: Southern blot analysis of EcoRI digested tail DNA from two F1 pups using an external *Rosa26*-specific hybridization probe. Control – DNA from a *Rosa26* knock-in mouse generated from ES cells, C57BL/6 – wildtype control

To enable gene editing by CRISPR/Cas9 in vivo*,* we aimed for germ line integration of a conditional Cas9 transgene into the *Rosa26* locus of C57BL/6 mice such that the delivery of the large Cas9 coding region into primary cells can be avoided. As a template for HDR, we constructed the targeting vector pRosa-Cas9, harboring an 11 kb insert flanked by standard *Rosa26* homology regions, extending 1 kb upstream and 4 kb downstream from the XbaI site mentioned above (Fig. 2a). The vector’s insert includes a CAG promoter region, a loxP-flanked transcriptional termination (Lox-Stop-Lox; LSL) element and the Cas9 coding region linked to an IRES-GFP reporter element. In addition, splice acceptor and polyA elements were placed upstream of the CAG promoter for the termination of the endogenous *Rosa26* transcripts (Fig. 2a). From pronuclear microinjections and transfer of 207 C57BL/6 zygotes with pRosa-Cas9 DNA, sgRosa26-1 and Cas9-162A RNAs we obtained 38 live pups (Table 1). To verify the activity of Cas9 in microinjected zygotes, these mice were first analyzed for the incidence of small deletions at the *Rosa26* target site. PCR amplification of the target region on genomic DNA from ear biopsies using the primer pair R26F2/R2 and the XbaI digestion assay confirmed the presence of XbaI resistant, NHEJ processed *Rosa26* alleles in 28 of 38 pups (74 %) (Fig. 2b). Next, we used a Cas9-specific primer pair for PCR and identified six mice harboring the Cas9 transgene (Fig. 2c). These potential founder mutants were further analysed to discriminate knock-in alleles from random vector integrations. None of these mice showed knock-in to both *Rosa26* alleles since additional wildtype or XbaI resistant PCR products were detected using the R26F2/SAR/R2 or F2/R2 primer combinations (Fig. 2c). For the detection of correct, targeted integrations by PCR, we used the R26F3 primer, recognizing a genomic sequence outside of the upstream homology region of the targeting vector, together with the vector specific primer SAR. The predicted 1.38 kb PCR product could be amplified from five of the six Cas9 transgenic mice, indicating the correct configuration of the knock-in allele in founders #18, #20, #35, #36 and #39 (Fig. 2d). Sequence analysis of these PCR products confirmed their identity as being derived from Rosa26^LSL-Cas9^ HDR alleles (Additional file 1: Figure S2). In 4 of 5 founders, Southern blot analysis of EcoRI digested tail DNA using a *Rosa26-*specific 5′-hybridization probe showed the predicted 6.0 kb band and thus correctly targeted alleles, whereas founder #20 exhibited a larger band, in addition to the 15.6 kb fragment from the *Rosa26* wildtype locus (Fig. 2e). For germline transmission of the targeted alleles, founders #18, 35, 36 and 39 were crossed to C57BL/6 wildtype mice and their offspring were genotyped using the Cas9 internal Cas9F/R primer pair. All founders transmitted the Rosa26^LSL-Cas9^ allele to about half of their offspring (Fig. 2f, Table 2). The *Rosa26* loci of one pup each from founder #18 (#18-40) and #39 (#39-22) were further analyzed by Southern blotting of EcoRI digested genomic DNA using an external *Rosa26* 5′ hybridization probe. Both pups showed the expected 6.0 kb band for the heterozygous Rosa26^LSL-Cas9^ allele, in addition to the 15.6 kb band derived from the *Rosa26* wildtype locus (Fig. 2g).

**Table 1:** Knock-in into the mouse *Rosa26* locus using sgRNA and Cas9 mRNA

Donor vector	Concentration (ng/μl)	Injected zygotes	Transferred embryos	Live pups (%)	Deletion alleles (%)	Knock-in alleles (%)
Rosa26^LSL-Cas9^	10	105	60	7 (12)	3 (43)	0 (0)
	20	183	147	31 (21)	25 (80)	5 (16)
Rosa26^LSL-Lgals-Cd274^	20	142	96	10 (10)	ND	2 (20)

**Table 2:** Germline transmission of Rosa26^LSL-Cas9^ alleles

Rosa26^LSL-Cas9^ founder	# pups	Male	Female	Rosa26^LSL-Cas9^ positive (%)
#18 (female)	9	7	2	5 (55)
#35 (male	15	8	7	10 (66)
#36 (male)	8	4	4	5 (47)
#39 (male)	17	8	9	7 (41)

Thus, using Cas9 and sgRosa26-1 RNAs, we achieved the targeted integration of an 11 kb conditional Cas9 transgene into the *Rosa26* locus of C57BL/6 zygotes at a frequency of 13 % and the Rosa26^LSL-Cas9^ founder mutants transmitted the targeted allele through their germ line.

### Cas9 is functional in B cells of Rosa26^LSL-Cas9^ mice

**Fig. 3:**
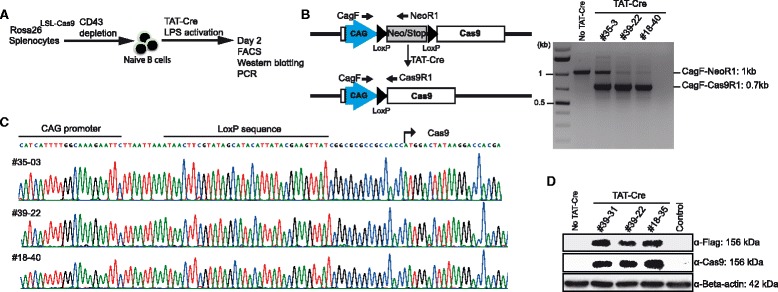
Cas9 is expressed in B cells of Rosa26^LSL-Cas9^ knock-in mice. **a**: Strategy for isolating naive B cells from spleens of three Rosa26^LSL-Cas9^ F1 mice by CD43 depletion and activation of Cas9 expression by deletion of the loxP flanked stop element upon treatment with TAT-Cre protein. **b**: Scheme of the TAT-Cre-mediated deletion of the Neo/Stop element (left). Detection of Cre-mediated deletion of the Neo/STOP cassette by PCR using DNA of TAT-Cre/LPS-treated B cells and the indicated primers. Rosa26^LSL-Cas9^ alleles produce a 1.0 kb band (CAGF-NeoR1 primers), Cre recombined alleles are detected by a 0.7 kb band (CAGF/Cas9R1 primers). **c**: Sequencing results of 0.7 kb PCR products (from **b**) showing the correct deletion of the loxP flanked stop element, leaving one loxP site in between the CAG promoter and the Cas9 coding region. **d**: Western blot analysis of lysates prepared from TAT-Cre/LPS treated B cells of three Rosa26^LSL-Cas9^ F1 mice using antibodies against the Flag Tag, Cas9, or Beta-actin

To confirm the functionality of the Rosa26^LSL-Cas9^ allele, we isolated naive B cells from spleens of three heterozygous F_1_ mice by using CD43 microbeads because the CD43 antigen is expressed on nearly all mouse leukocytes except for immature and resting mature B cells. The B cells were treated with cell permeable Tat-Cre recombinase for deletion of the loxP-flanked stop element, activated with LPS, inducing B cell proliferation and differentiation, for 2 days. The activated B cells were harvested and used for isolation of genomic DNA and cellular proteins (Fig. 3a). As shown by a three primer PCR for the detection of the recombined alleles, Tat-Cre removed the stop element with high efficiency (Fig. 3b) and sequence analysis of the PCR products confirmed the presence of a single loxP site between the CAG promoter and the Cas9 coding region (Fig. 3c). The expression of Cas9 protein from the activated Rosa26^LSL-Cas9^ allele was analyzed by Western blotting using lysates of Tat-Cre treated B cells and Cas9 or Flag-Tag specific antibodies. Both antibodies verified the expression of the 156 kD Cas9 protein in Tat-Cre treated B cells from three heterozygous Rosa26^LSL-Cas9^ mice (Fig. 3d).

**Fig. 4:**
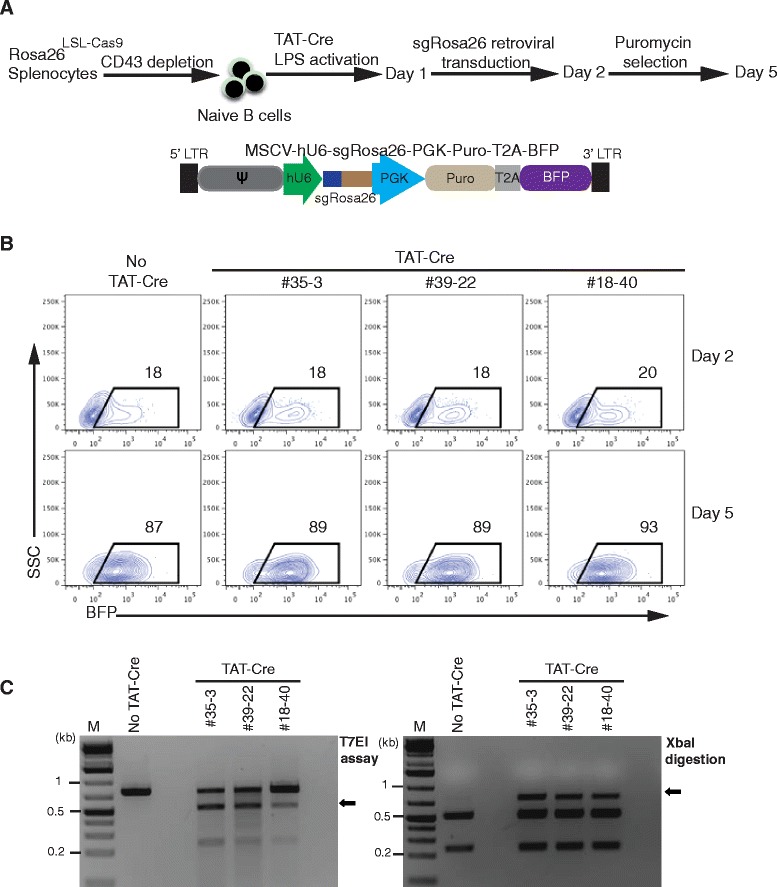
Cas9 is functional in B cells of Rosa26^LSL-Cas9^ knock-in mice. **a**: Scheme of genome editing in primary mouse B cells using CRISPR/Cas9. Naive B cells from spleens of three individual heterozygous Rosa26^LSL-Cas9^ F1 mice were isolated using CD43 depletion, treated with TAT-Cre and stimulated with LPS for 24 h. TAT-Cre/LPS treated B cells were transduced with retroviral particles co-expressing sgRosa26-1 and BFP to target the *Rosa26* locus. One day later, the transduced B cells were selected with puromycin until day 5. **b**: FACS analysis of B cells (from **a**) before (day 2) and after puromycin selection (day 5). The gate indicates the fraction (percentage) of successfully transduced BFP^+^ cells. **c**: Gel electrophoresis of T7EI or XbaI digested PCR products (R26T7F/R26T7R primers) amplified from DNA of FACS sorted BFP^+^ cells (from **b**), indicating sequence deletions by the presence of T7EI sensitive or XbaI resistant bands (arrows)

The nuclease activity of the expressed Cas9 protein was confirmed by the transduction of Tat-Cre treated, LPS activated B cells with retroviral particles expressing sgRosa26-1, a puromycin resistance and a BFP gene (Fig. 4a). The transduced B cells of F1 Rosa26^LSL-Cas9^ heterozygous pups from three different founders (#18, #35 and #39) were selected with puromycin for three days, leading to an enrichment of BFP^+^ transduced cells to 90 % (Fig. 4b). We then isolated genomic DNA from FACS sorted BFP^+^ cells from the experimental and control cultures and performed PCR amplification of the sgRosa26-1 target region, followed by XbaI digestion and T7EI assays. In both groups, we found high levels of XbaI resistant and T7E sensitive *Rosa26* PCR products (Fig. 4c), indicating sufficient Cas9 expression from the conditional Rosa26^LSL-Cas9^ allele to achieve targeted mutagenesis.

These results verified the expression of functional Cas9 nuclease in Rosa26^LSL-Cas9^ F_1_ offspring upon Cre-mediated activation of the transgene. Since the targeted allele was introduced into C57BL/6 zygotes, the Rosa26^LSL-Cas9^ mouse line allows Cas9-mediated gene editing in vivo and in primary cells of C57BL/6 mice. The Rosa26^LSL-Cas9^ line will be distributed through the Jackson laboratory (www.jax.org).

### Analysis of off-target activity

**Fig. 5:**
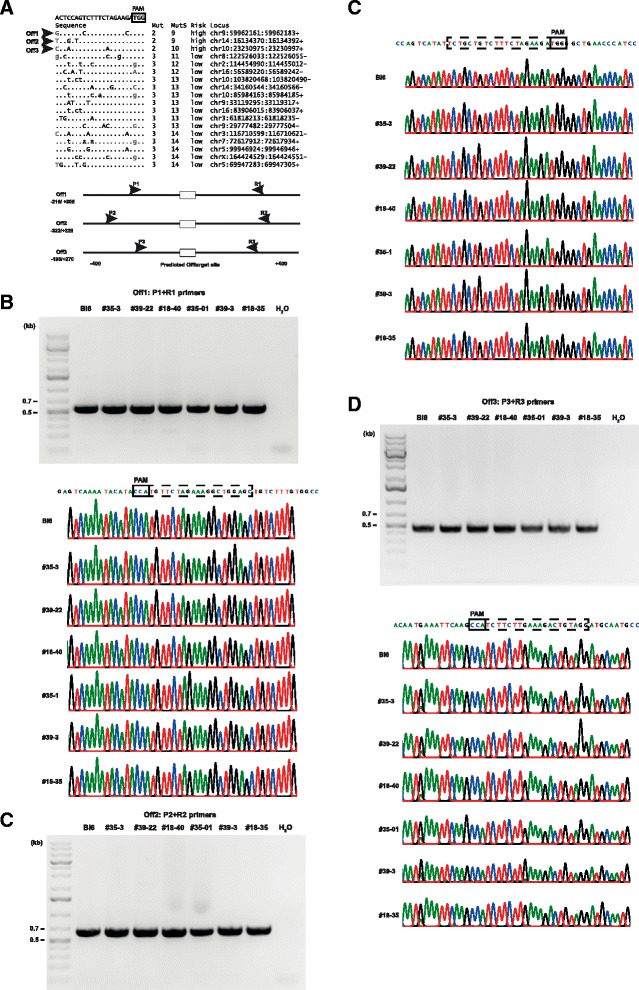
Analysis of off-target activity. **a**: The top 18 predicted off-target sites of the Rosa26-1 target sequence sorted according to sequence divergence (upper panel) and the PCR scheme for the analysis of the top 3 off-targets (lower panel). Negligible mismatches are shown in grey. **b**: PCR amplification of the off-target site 1 (Off1) from two F1 pups each derived from the mutant founders #18, #35 or #39 (upper panel) and sequencing results of the respective bands (lower panel, Bl6-C57Bl/6 wildtype control). **c**: PCR amplification of the off-target site 2 (Off2) from two F1 pups each derived from the mutant founders #18, #35 or #39 (upper panel) and sequencing results of the respective bands (lower panel, Bl6-C57Bl/6 wildtype control). **d**: PCR amplification of the off-target site 3 (Off3) from two F1 pups each derived from the mutant founders #18, #35 or #39 (upper panel) and sequencing results of the respective bands (lower panel, Bl6-C57Bl/6 wildtype control)

Genomic sequences showing high similarity to the sgRosa26-1 target-sequence may lead to unintended gene editing at such off-target sites. To determine the importance of off-target modification in our system, we predicted the off-target sites of sgRosa26-1 in the mouse genome based on sequence similarity to its target-sequence and selected the three sites with the highest risk of being edited (Fig. 5a). We then amplified and sequenced these loci from six heterozygous Rosa26^LSL-Cas9^ F_1_-mice (derived from the founder mutants #18, #35 and #39) and from the six Cas9 positive mice of the founder generation (#18, #20, #35, #36, #39, #43). We did not detect any genetic modification in all of the analysed off-target loci, since solely wildtype, but no mixed sequence reads were obtained (Fig. 5b-d, Additional file 1: Figure S3). Although we cannot rule out off-target effects in other loci, these results suggest that in the present setting, off-target effects are not dominant.

### Knock-in of a conditional Galectin-1-E2A-PD-L1 transgene into *Rosa26* of C57BL/6 zygotes

**Fig. 6:**
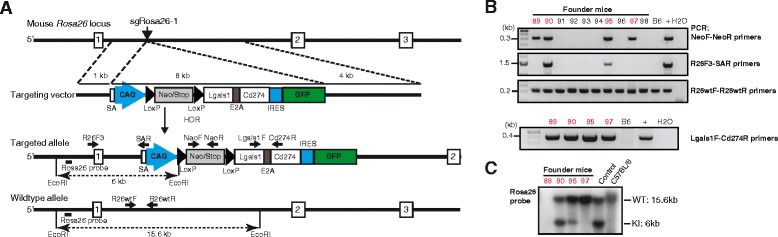
Knock-in of a conditional Galectin-1-E2A-PDL1 transgene into *Rosa26* of C57BL/6 zygotes. **a**: Strategy for insertion of the CAG-loxPSTOPloxP-Lgals1-E2A-Cd274-IRES-EGFP cassette into the mouse *Rosa26* locus. sgRosa26-1 and Cas9 introduce a double-strand break between 1 kb and 4 kb fragments used as homology arms in the targeting vector. Homology-directed repair (HDR) leads to the insertion of the cassette into the genome. The locations of PCR primers, restriction sites and the *Rosa26* hybridisation probe in the targeted and wildtype alleles are indicated. **b**: Gel electrophoresis of PCR reactions from genomic DNA of ten pups derived from microinjections using primers NeoF/R for detection of an internal vector segment (stop element, top). Second panel: Mouse DNAs were further tested for correct knock-in (KI) into *Rosa26* using a PCR with a forward primer located outside of the 5′-homology region (R26F3) and a reverse primer located in transgene (SAR); the predicted band has a size of 1.38 kb. Third panel: DNA quality was controlled with a *Rosa26*-specific PCR (R26wtF/R primers, 0.2 kb). Lower panel: PCR detection of the Galectin-1-E2A-PDL1 transgene using Lgals1F (forward) and Cd274R (reverse) primers. +: Positive control DNA from a *Rosa26* knock-in mouse generated from ES cells; H_2_O: negative control. **c**: Southern blot analysis of EcoRI digested tail DNA from vector-positive mice (from B) using an external *Rosa26*-specific hybridization probe. Knock-in alleles are predicted to show a 6 kb EcoRI band; for sample #89 the tail biopsy yielded insufficient gDNA. Control – DNA from a *Rosa26* knock-in mouse generated from ES cells, C57BL/6 – wildtype control

Evidence that the *Rosa26* targeting strategy outlined above can be extended to other transgenes was obtained in experiments aiming at the generation of signal-on alleles encoding the immunomodulatory proteins Galectin-1 (Lgals1) and PD-L1 (Cd274), linked by a self-cleaving E2A peptide. For this purpose we constructed a *Rosa26* targeting vector by inserting the 8 kb transgene into a Gateway cloning destination vector [16], harboring λ-phage attR sites in between the loxP flanked stop element and an IRES-GFP reporter gene (Fig. 6a). For knock-in into the *Rosa26* locus the Lgals1-E2A-Cd274 targeting vector was microinjected together with sgRosa26-1 (12.5 ng/μl) and Cas9-162A (25 ng/μl) RNAs into the pronuclei of 142 C57BL/6 zygotes. To control for embryo viability, the injected zygotes were cultured overnight to the 2-cell stage. We recovered 96 such embryos (68 %) that, upon transfer into foster mothers, resulted in the birth of 10 pups (Table 1). Genomic DNA from ear biopsies was first used for the detection of vector integrations by PCR amplification of an internal segment of the stop element using the NeoF/R primer pair (Fig. 6b). Four pups showed the predicted 324 bp band and were further genotyped using the R26F3/SAR primer pair, enabling the detection of *Rosa26* HDR alleles by a forward primer (F3) outside of the vector’s upstream homology region and a vector-specific reverse primer (SAR). The predicted 1.38 kb PCR product could be amplified from 2 pups (#90 and #95), confirming the presence of *Rosa26* knock-in alleles (Fig. 6b). The presence of the protein coding segment was further confirmed using an Lgals1 and Cd274 specific primer pair. In addition, we verified the correct integration of the targeting vector in both founders by Southern blot analysis of EcoRI digested tail DNA using an external *Rosa26* 5′ hybridization probe. Both founders showed the 6.0 kb band predicted for the Rosa26^Lgals-Cd274^ allele, in addition to the 15.6 kb band derived from the *Rosa26* wildtype locus (Fig. 6b). The two founder mutants are presently bred for germline transmission of the targeted Rosa26^LSL-Lgals/Cd274^ allele.

In conclusion, the present direct targeting approach using CRISPR/Cas9 in zygotes allows the rapid generation of new *Rosa26* knock-in mouse lines on the C57BL/6 inbred background. Since 10–17 % of transferred embryos developed into live pups and 10–20 % of them were correctly targeted mutants, the establishment of a new knock-in line requires the microinjection and transfer of no more than 100–200 zygotes.

### Resources for *Rosa26* targeting in zygotes

**Fig. 7:**
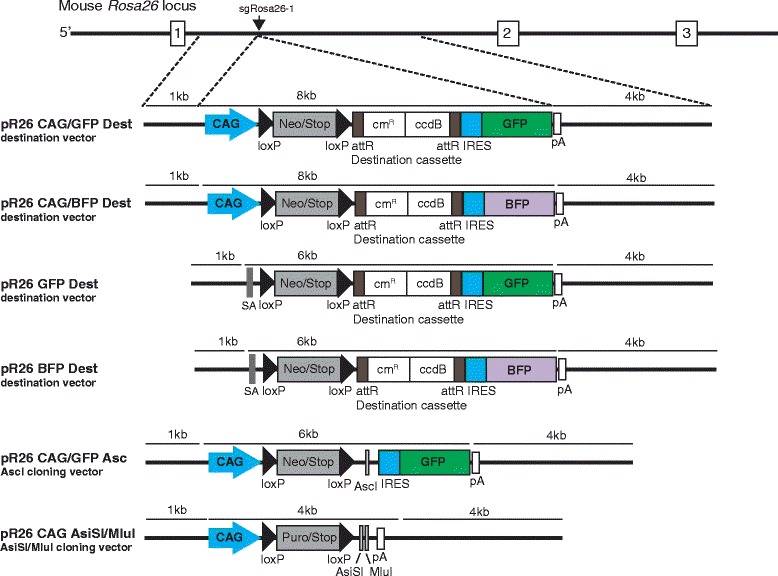
*Rosa26* targeting vectors for conditional gene expression. For the construction of new conditional *Rosa26* targeting vectors we provide plasmids which include the standard *Rosa26* homology regions of 1 kb and 4 kb merging at the sgRosa26-1 target site and a loxP-flanked stop element containing a neomycin (Neo) or puromycin (puro) resistance gene. Gene expression is either driven by the CAG promoter (CAG) or through the *Rosa26* promoter by (pR26 GFP, pR26 BFP) capture of the endogenous transcript via a splice acceptor site (SA). Coding regions for transgene expression can be inserted into destinations vectors for Gateway cloning by replacement of the λ phage attR-flanked cm^R^/ccdB segment or into unique AscI or AsiSI/MluI restriction sites. For the imaging of gene expression most vectors, except pR26 CAG AsiSI/MluI, include a GFP or BFP reporter linked with an IRES element. pA –polyadenylation site

To facilitate the construction of new conditional *Rosa26* targeting vectors, we provide targeting vectors harboring pairs of λ-attR sites for the insertion of coding regions in between a loxP flanked stop element and an IRES-GFP or -BFP reporter by Gateway cloning (Fig. 7). Each of these versions either contains the CAG promoter for transgene expression or an acceptor element for splicing to the endogenous *Rosa26* transcript. In addition, we provide conditional targeting vectors enabling the standard cloning of new inserts into an AscI site located upstream of an IRES-GFP reporter or into the AsiSI or MluI site of a reporter-free plasmid (Fig. 7). For the preparation of sgRosa26-1 and Cas9-162A RNAs for microinjection we provide plasmids pBS-U6-sgRosa26-1 and pCAG-Cas9-162A. All plasmids will be distributed via the Addgene repository (www.addgene.org) and protocols for RNA production and the PCR-based detection of modified *Rosa26* alleles and off-target analysis are included in the Additional file 2.

**Fig. 8:**
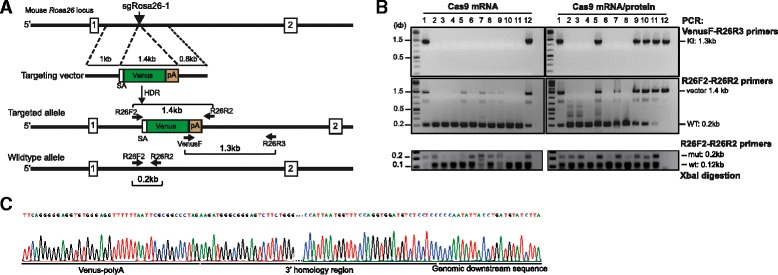
Coinjection of Cas9 mRNA and Cas9 protein into zygotes. **a** Strategy for insertion of a Venus reporter into the mouse *Rosa26* locus. sgRosa26-1 and Cas9 introduce a double-strand break between 1 kb and 0.8 kb fragments used as homology arms in the pR26-Venus targeting vector. The locations of PCR primers in the targeted and wildtype *Rosa26* alleles are indicated. SA- splice acceptor, pA polyadenylation site. **b** Mouse zygotes were microinjected with pR26-Venus, sgRosa26-1 and Cas9 mRNA or Cas9 mRNA and protein. The embryos were cultured for 4 days and genomic DNA was isolated from 12 blastocysts each, for PCR-based detection of HDR or deletion events. Top panel: gel electrophoresis of PCR products. Targeted alleles (KI) are detected by amplification of a 1.3 kb genomic segment using the vector-specific primer VenusF and the R26R3 primer, located downstream of the vector homology region. The presence of integrated or nonintegrated vector DNA was tested using the R26F2/R2 primer pair, amplifying a 1.4 kb vector segment as well as 0.2 kb of the *Rosa26* target region (middle panel). Lower panel: *Rosa26* alleles with sequence deletions were detected by 0.2 kb of the target region (R26F2/R26R2 primers), followed by XbaI digestion and gel separation. XbaI resistant PCR products indicate the presence of sequence deletions (mut, 0.2 kb) whereas wildtype products are reduced to 0.12 kb fragments (wt). **c** Sequencing of PCR products amplified with primers VenusF and R26R3 (from B, top) showed the predicted recombination between the targeting vectors homology region and adjacent downstream genomic sequence

In addition to the standard conditions with sgRosa26-1 and Cas9-162A RNAs, as used for the generation of Rosa26^LSL-Cas9^ and Rosa26^LSL-Lgals/Cd274^ mice, we explored whether the frequency of knock-in events can be further increased by the co-injection of recombinant Cas9 protein. For these test experiments we cultured microinjected zygotes to the blastocyst stage, extracted genomic DNA and determined the frequency of *Rosa26* knock-in and deletion events by PCR. For the knock-in into *Rosa26,* we used a Venus targeting vector harboring the 1 kb standard 5′-homology region and a 3′-homology region shortened from 4 kb to 0.8 kb (Fig. 8a) to facilitate the detection of recombined alleles by PCR using the vector-specific primer VenusF and the external downstream R26R3 primer (Table 3). We analyzed two groups of 12 blastocysts each that were derived from the microinjection of zygotes with aliquots of an identical preparation of sgRosa26-1 RNA, Cas9-162A mRNA and pRosa26-Venus DNA, except that one sample was supplemented with Cas9 protein (30 ng/μl) immediately before injection. As shown in Fig. 8b, we found two embryos (17 %) positive for the 1.38 kb knock-in PCR product in the group microinjected with the RNA/DNA preparation alone, comparable to our previous results. In the group microinjected with additional Cas9 protein 6 of 12 embryos (50 %) were positive for the knock-in PCR product. Although the small sample size prevents statistical evaluation, it is possible that the combined use of Cas9 mRNA and protein leads to increased Cas9 cleavage and improved HDR. The frequency of small deletions at the *Rosa26* target site, as assessed by XbaI digestion of PCR products, was also clearly elevated in embryos co-injected with Cas9 protein (Fig. 6b).

**Table 3:** PCR primers used in this study

PCR type	Primer	Sequence (5′ > 3′)
Nested PCR for Rosa26 locus	R26F1	CCAAAGTCGCTCTGAGTTGTTATCAGT
	R26R1	GGAGCGGGAGAAATGGATATGAAG
	R26F2	GCCTCCTGGCTTCTGAGGACCG
	R26R2	TCTGTGGGAAGTCTTGTCCCTCC
Cas9 transgene	Cas9F	GGCATCCTGCAGACAGTGAAGGTGG
	Cas9R	CGGTTCTTGTCGCTTCTGGTCAGCA
Homozygous		
Heterozygous for R26-Cas9 allele	R26F2	GCCTCCTGGCTTCTGAGGACCG
	R26R2	TCTGTGGGAAGTCTTGTCCCTCC
	SAR	CCTGGACTACTGCGCCCTACAGA
Long PCR for correct		
Integration	R26F3	CTGCCCGAGCGGAAACGCCACTGAC
	SAR	CCTGGACTACTGCGCCCTACAGA
Neo transgene	NeoF	GCTAACCATGTTCATGCCTTC
	NeoR	CGTTGGCTACCCGTGATATT
Loading PCR for Rosa26 locus	R26wtF	GGAGTGTTGCAATACCTTTCTGGGAGTTC
	R26wtR	TGTCCCTCCAATTTTACACCTGTTCAATTC
Lgals-Cd274 transgene	Lgals1F	CAAGATTAAGTGCGTGGCC
	Cd274R	CATTTCCCTTCAAAAGCTGGTC
Venus correct integration	VenusF	GGCCTCTCGAGCCTCTAGAACTATAGTG
	R26R3	CAAGCTCACAAGACCTTAGGTCAGGA
LoxP-flanked Stop cassette deletion	CagF	CAAGCTCACAAGACCTTAGGTCAGGA
	NeoR1	ATGGGATCGGCCATTGAACAAGATGG
	Cas9R1	CGGCCTTGTCGGTGCTGTCCACCAG
PCR Rosa26 for T7EI and RLFP	R26T7F	CGTGCAAGTTGAGTCCATCCGCC
	R26T7R	ACTCCGAGGCGGATCACAAGCA

## Discussion

Efficient gene editing in mouse zygotes using the CRISPR/Cas9 system has been mainly reported for the generation of knockout alleles by small sequence deletions in genetically hybrid embryos. However, many research applications require precisely targeted mutations on the C57BL/6 inbred background. Specifically, targeted insertions into the *Rosa26* locus are frequently used for the constitutive or conditional expression of transgenes in a standardized single copy configuration. Here we established a CRISPR/Cas9 based approach for generating *Rosa26* knock-in alleles in C57BL/6 zygotes. We found that transgene inserts of 8 or 11 kb were targeted to *Rosa26* in 10–20 % of the mice derived from microinjections of 100–200 C57BL/6 zygotes. For the detection of potential off-target modifications by Cas9, we tested six Rosa26^LSL-Cas9^ F_1_ pups each at three predicted off-target sites. Since these sites showed no modifications, we conclude that sgRosa26-1 does not lead to obvious, frequent off-target processing.

To facilitate the future production of *Rosa26* knock-in mouse lines, we provide various targeting constructs for the insertion of new transgenes using Gateway or restriction site cloning. Furthermore, we found a higher proportion of *Rosa26* knock-in alleles in a small group of embryos microinjected with Cas9 mRNA and additional Cas9 protein. Although this result is out of reliable statistical evaluation, it is possible that the microinjection of Cas9 protein and sgRNA stimulates DSB formation and HDR early on, complementing and preceding the translation of Cas9 mRNA that supports sustained nuclease activity over time. Thus, provided that live mutants will be obtained at similar rates, the combined supply of Cas9 mRNA and protein could further streamline the production of *Rosa26* and other knock-in mutants, provided that future experiments will confirm our initial findings. In addition, the suppression of NHEJ repair by inhibition of DNA Ligase IV [13, 17] may provide another option to increase the yield of *Rosa26* HDR alleles in zygotes. Since we also successfully used a shortened, 0.8 kb 3′-homology region for the knock-in of Venus into *Rosa26*, it will be interesting to further investigate which lengths of homology regions, in relation to the size of transgene inserts, are required for optimal HDR efficiency.

Previous studies on the direct targeting of *Rosa26* used pairs of zinc-finger nucleases [7] or TALEN [18] in zygotes and achieved HDR frequencies of 1.7–4.5 % (ZFN) or 5.8–11.7 % (TALEN), respectively, for the insertion of reporter genes. Intrinsic limitations for DSB induction by these earlier nuclease designs may be posed by the requirement for binding of two protein molecules to the target DNA and for the dimerization of their nuclease domains. Since for Cas9, DSBs are formed by only one single protein/RNA complex we reason that higher levels of mutagenesis can be achieved by the delivery of preformed Cas9/sgRNA into zygotes. As an alternative to sequence-specific nucleases, Cre/loxP mediated recombination has been used in zygotes for vector integrations by recombinase-mediated cassette exchange (RMCE) into a modified *Rosa26* allele at efficiencies of 4.4–25 % [19, 20]. However, the RMCE approach is incompatible with the use of Cre/loxP dependent constructs and requires the maintenance of a breeding colony of RMCE acceptor mice for embryo production.

Using CRISPR/Cas9 we generated a new conditional Cas9 mouse line for in vivo and ex vivo gene editing in the C57BL/6 inbred background. A similar strain was previously generated by gene targeting in 129-derived R1 ES cells [21]. A minimum of 10 backcross cycles with C57BL/6 mice will be required for the establishment of a congenic Cas9 strain, precluding prompt phenotypic studies that require this widely used inbred background. Since *Rosa26* has been also validated as a ‘safe harbor’ integration site in rats and pigs using ES cells [22] or nuclear transfer [23], CRISPR/Cas9-based *Rosa26* knock-in in zygotes could also be of use in these species. Finally, efficient targeting of the *Rosa26* locus in the C57BL/6 background allows the direct targeting of complex experimental compound mutants and thus bypassing time-consuming breeding strategies.

## Conclusions

*Rosa26* is frequently used as standardized insertion site for single transgene copies via gene targeting in ES cells, an approach that requires laborious handling of cell cultures and 9–12 months’ time until the identification of positive F_1_ pups. Here we applied CRISPR/Cas9-assisted mutagenesis for the single step insertion of conditional transgenes into *Rosa26* of C57BL/6 zygotes. Using modified Cas9 mRNA and sgRNA targeting the intronic XbaI site of *Rosa26*, we achieved the knock-in of 8–11 kb inserts in 10–20 % of pups derived from microinjections of C57BL/6 embryos. Upon the combined microinjection of Cas9 mRNA and Cas9 protein we found knock-in alleles in 50 % of cultured blastocysts. For modifying gene function by CRISPR/Cas9 in somatic tissues of C57BL/6 mice, to avoid the vector-mediated delivery of the large Cas9 transgene into primary cells, we generated *Rosa26* knock-in mice for the Cre/loxP-dependent expression of Cas9. Taken together, our protocols and resources support the fast and direct generation of new *Rosa26* knock-in alleles and of Cas9-mediated in vivo gene editing in the widely used C57BL/6 inbred strain.

## Methods

### Cloning of targeting vectors

Targeting vectors were cloned by modifying a published *Rosa26* targeting vector containing a loxP-flanked STOP cassette and an IRES-GFP reporter [24]. A CAG promoter, preceded by two copies of the bovine growth hormone gene poly(A)-addition signal, was inserted upstream of the STOP cassette using a PacI site as previously published [25]. A diphtheria toxin gene downstream of the 3′ homology arm was removed using AgeI and AsisI restriction sites. cDNA coding for Cas9 was amplified from plasmid pX330 (Addgene #42230) and was inserted into the targeting vector using an AscI restriction site. Lgals1 cDNA was amplified from sequence NM_008495.2, Cd274 cDNA was amplified from sequence NM_021893.3 (both from plasmids provided by the DNA Resource Core at Harvard Medical School) and the published sequence coding for E2A was purchased as DNA oligonucleotides [26]. Lgals1-E2A-Cd274 was assembled by overlapping PCR and cloned into a Gateway entry vector using the pENTR/D-TOPO Cloning Kit (Invitrogen). To convert the *Rosa26* targeting vector into a destination plasmid, the AscI restriction site was used to insert a destination cassette for Gateway cloning with the Gateway Vector Conversion System (Invitrogen). Lgals1-E2A-Cd274 cDNA was then transferred into the destination cassette of the targeting vector using Gateway LR Clonase Enzyme Mix (Invitrogen).

### Microinjection of zygotes

Cas9 mRNA was prepared in a single step by in vitro transcription from plasmid pCAG-Cas9-162A [14] linearized with AsiSI, AscI and XbaI using the mMessage mMachine T7 Ultra kit (Life Technologies, Ambion, AM1345, Life Technologies, Carlsbad, USA) (omitting the polyadenylation step) and the MEGAclear kit (Ambion, #1908). To produce the template for sgRNA in vitro transcription, sgRosa26-1 was amplified by PCR from plasmid pX330-sgRosa26-1-T2A-BFP (Addgene #64216) [13] with the forward primer T7-sgRosa26-for (5′-TTAATACGACTCACTATAGGACTCCAGTCTTTCTAGAAGAGT) and the reverse primer T7-sgRNArev (5′-AAAAGCACCGACTCGGTGCC). One microgram template DNA was used for in vitro transcription using the Megashortscript kit (Ambion, #AM1354) followed by the MEGAclear kit for RNA purification. The quality of mRNAs was controlled by agarose gel electrophoresis under denaturing conditions using the NorthernMax-Gly system and the RNA Millenium size marker (Life Technologies). RNAs and targeting vectors were diluted in microinjection buffer (10 mM Tris, 0.1 mM EDTA, pH 7.2) to the indicated working concentrations, filtrated through a centrifugal filter (Ultrafree, PFTE, Millipore, cat. no. UFC30LG25) and stored in single use aliquots at −80 °C. Where indicated, Cas9 protein (ToolGen Inc, Seoul, South Corea) was supplemented immediately before injection. Step-by-step protocols for RNA and sample preparation are included as Supplementary Methods in the Additional file 2.

For microinjections, zygotes were obtained by mating of C57BL/6 N males with super-ovulated C57BL/6 N females (Charles River, Sulzbach, Germany) using standard procedures [27]. Zygotes were microinjected into one pronucleus as previously described [28]. Injected zygotes were transferred into the oviducts of pseudo-pregnant NMRI female mice to obtain live pups. All mice showed normal development and appeared healthy. Mice were handled according to institutional guidelines and all experiments were performed under registration and ethical approvement (Registration No. IC10b-G0162/12) by the *Landesamt für Gesundheit and Soziales* of the federal state of Berlin (Turmstr. 21, 10559 Berlin, Germany) Mice were housed in individually ventilated cages (IVC, Tecniplast) in a specific pathogen-free facility on a 12 h light/dark cycle with *ad libitum* access to food and water.

### PCR, T7EI and RLFP assays

Genomic DNA from blastocysts was extracted using the QuickExtract DNA extraction kit (Epicentre) following the manufacturer’s instructions. PCR was performed using Herculase II Fusion DNA Polymerase (Agilent Technology) with gene-specific primers. For the T7EI assay, the PCR product was cleaned up and digested with T7EI (New England Biolabs following the manufacturer’s instructions. For the RLFP assay, PCR products were digested with the restriction enzyme XbaI (Thermo Scientific). Cleaved DNA fragments were separated on 2 % agarose gels and the DNA concentration of each band was quantified using the ImageJ software. Percent values of indels were calculated as described [29]. For genotyping by PCR, serial primer pairs were used as listed below.

### DNA sequencing

Specific PCR products were cleaned up and directly sequenced by the Sanger method (LGCgenomics, Berlin, Germany). In addition, the DNA fragments were cloned into pSTblue-1 Blunt vector (Novagen), plasmids were isolated using the NucleoSpin Plasmid kit (Macherey-Nagel). Plasmids were sequenced using T7 forward primer.

### Analysis of off-target sites

The Rosa26-protospacer (ACTCCAGTCTTTCTAGAAGATGG) was aligned to the mouse genome (mm9) using BWA (0.7.12) [30]. Off-target sites were evaluated using an in-house developed tool for protospacer-design. The primers used for PCR-ampflication were P1 (5′-TTGGTTCCCAACACTCACAG-3′), R1 (5′-TGTGTAACTGCTCTGTTGTCTCC-3′), P2 (5′-CTTTGGGTTCCCTCAGTAGAAG-3′), R2 (5′-AAGACCCAAACAGGTATGCAG-3′), P3 (5′-CCACAGGGATAGGCAATAAAGA-3′) and R3 (5′-GCTGAGCTGTCCCAATGAGT-3′). PCR products were sequenced by the Sanger method (LGCgenomics, Berlin, Germany) using R1, P2 and R3 for Off1, Off2 and Off3, respectively.

### Southern blotting

Southern blotting for correct integration of the targeting construct into the *Rosa26* locus was done as described [24]. Briefly, genomic DNA was isolated from tails and 10 μg were digested with EcoRI. DNA fragments were separated on a 0.7 % agarose gel. The gel was washed two times for 5 min in dH_2_O, incubated two times for 15 min in 0.125 M HCl for depurination, washed two times for 5 min in H_2_O, and was finally denatured by incubating two times for 15 min in 0.5 M NaOH/1.5 M NaCl. DNA was blotted over night to a Hybond XL membrane (GE Healthcare). The membrane was then neutralized for 10 min in 0.5 M Tris–HCl pH 7.2/1 M NaCl, dried, and UV-crosslinked by irradiation with 120,000 μJ cm^−2^. Then, the membrane was pre-incubated for 3 h at 65 °C in ExpressHyb Hybridization Solution (Takara). 25 ng probe was radioactively labeled using Ladderman Labeling Kit (Takara) by adding 25 μCi ^32^P-dCTP and purified on a Sephadex-G50 column (GE Healthcare). The probe was first denatured and then incubated with the membrane over night at 65 °C. Next, the membrane was briefly washed twice in 2 × SSC/1 % SDS, then incubated in 2 × SSC / 1 % SDS for 30 min, then incubated in 1 × SSC / 1 % SDS for 30 min, followed by 0.5 × SSC / 1 % SDS for 30 min, all at 65 °C. The membrane was used to expose an X-ray film at −80 °C in the dark for 3 to 7 days.

### B cell culture, stimulation and TAT-Cre treatment

Naïve B cells from Rosa26^LSL-Cas9^ and C57BL/6 mice were isolated by CD43 depletion using CD43 microbeads (Miltenyi Biotec). B cells were cultured at 1x10^6^ cells/ml in DMEM medium supplied with 15 % FBS, 2 mM HEPES (Gibco), 2 mM Sodium Pyruvate (Gibco), 2 mM L-Glutamine (Gibco), and 1x NAA (Gibco), beta-mercapthoethanol (Sigma) and stimulated with LPS (10 μg/ml). In addition, 5–10x10^6^ naïve B cells isolated from Rosa26^LSL-Cas9^ mice were treated with TAT-Cre protein as previously described [31]. Briefly, CD43-depleted B cells were washed 3 times with HyClone™ ADCF-Mab medium (GE Heathcare), incubated with TAT-Cre for 45 min at 37 °C, finally the cells were washed with complete medium. TAT-Cre-treated B cells were stimulated with LPS for 2 or 3 days.

### Retroviral transduction

The MSCV plasmid expressing the sgRNA to target the mouse *Rosa26* locus was transfected into the packaging cell line Plat-E (Cell Biolabs) using Calcium phosphate protocol. 24 h after transfection, the medium was changed and the transfected cells were incubated at 32 °C. The viral supernatant was collected at 48 and 72 h after transfection. The supernatant was concentrated using Amicon Ultra-15 Centrifugal Filter (Merck) according to the manufacture’s protocol. 5 × 10^5^ of LPS-activated B cells were transduced with concentrated retroviral particles using spin transduction method. The reporter positive cells were quantified using a Fortessa cell analyzer (Becton Dickinson).

### FACS sorting and analysis

The BFP^+^ B cells were sorted into 15 ml Falcon tubes with complete medium, cells were centrifuged and genomic DNA was isolated. For flow cytometry analysis, B cells were harvested, washed 2x with cold PBS, resuspended in PBS/1 % BSA FACS buffer and stained with anti-mouse CD19 Brilliant Violet 605™ and B220 Brilliant Violet 785™ (Biolegend). The stained cells were analysed with a Fortessa cell analyzer (Becton Dickinson). Dead cells were excluded by DAPI (Sigma).

### Western blot analysis

Naive B cells were treated with TAT-Cre protein, and stimulated with LPS (10 μg/ml) for 2 days. The protein lysates were isolated from the activated B cells as previously described [13] and separated by SDS-PAGE. Blots were probed with anti-Flag (M2, Sigma), anti-Cas9 (Novus Biologicals) and anti-beta-actin (AC-74, Sigma) antibodies. The probed blots were developed with secondary anti-mouse IgG HRP (eBioscience) and visualised using the ECL detection kit (GE Healthcare).

## Additional files

Additional file 1: Figure S1. CRISPR/Cas9 induced DSBs at the *Rosa26* intronic XbaI site in mouse zygotes; **Figure S2**: Sequence analysis of founder derived PCR products. **Figure S3**: Analysis of off-target activity. (DOC 1735 kb) Additional file 2:
**Supplementary Methods and Plasmid maps.** (PDF 358 kb)

## Abbreviations

DSB: double-strand break
ES cells: embryonic stem cells
HDR: homology directed repair, LSL, lox-stop-lox
NHEJ: non-homologous end joining
