# Supplementary material for: Efficient generation of Rosa26 knock-in mice using CRISPR/Cas9 in C57BL/6 zygotes
Source: BMC Biotechnol. 2016 Jan 16;16:4. doi: 10.1186/s12896-016-0234-4 (PMC4715285; doi:10.1186/s12896-016-0234-4)
Supplement: Additional file 1: Figure S1. — CRISPR/Cas9 induced DSBs at the Rosa26 intronic XbaI site in mouse zygotes; Figure S2: Sequence analysis of founder derived PCR products. Figure S3: Analysis of off-target activity. (DOC 1735 kb) [file 12896_2016_234_MOESM1_ESM.doc]

**Efficient generation of *Rosa26* knock-in mice using CRISPR/Cas9 in C57BL/6 zygotes**

Van Trung Chu, Timm Weber, Robin Graf, Thomas Sommermann, Kerstin Petsch, Ulrike Sack, Pavel Volchkov, Klaus Rajewsky, Ralf Kühn

**Additional file 1**

**Figure S1. CRISPR/Cas9 induced DSBs at the *Rosa26* intronic XbaI site in mouse zygotes.**

Agarose gel electrophoresis of XbaI digested PCR products amplified with the R26F2/R26R2 primer pair from blastocysts derived from zygotes microinjected with the indicated concentration of sgRosa26-1 and Cas9 RNAs. XbaI resistant 0.2 kb bands indicate the presence of modified *Rosa26* alleles harboring sequence deletions. M – size marker.

**Figure S 2. Sequence analysis of founder derived PCR products**

PCR products amplified from founders #18, #20, #35, #36 and #39 using the R26F3/SAR primer pair were sequenced and compared to the predicted sequence of the ^Rosa26LSL-Cas9^ allele. Shown are segments including both ends of the targeting vector`s upstream homology region.

**Figure S3. Analysis of off-target activity in Cas9 positive founder mice**

PCR amplification of the off-target sites (see Fig. 5) Off1 (**a**), Off2 (**b**) and Off3 (**c**) and sequencing results of the respective bands derived from the six Cas9 positive mice (Fig. 2D) of the founder generation (#18, #20, #35, #36, #39, #43). (H_2_O - water control).
