# Supplementary material for: Efficient generation of Rosa26 knock-in mice using CRISPR/Cas9 in C57BL/6 zygotes
Source: BMC Biotechnol. 2016 Jan 16;16:4. doi: 10.1186/s12896-016-0234-4 (PMC4715285; doi:10.1186/s12896-016-0234-4)
Supplement: Additional file 2: — Supplementary Methods and Plasmid maps. (PDF 358 kb) [file 12896_2016_234_MOESM2_ESM.pdf]

Efficient generation of *Rosa26* knock-in mice using CRISPR/Cas9 in C57BL/6 zygotes

Van Trung Chu, Timm Weber, Robin Graf, Thomas Sommermann, Kerstin Petsch, Ulrike Sack, Pavel Volchkov, Klaus Rajewsky, Ralf Kühn

Additional file 2

Supplementary Methods

1. Production of sgRNA
2. Production of Cas9-162A mRNA
3. Preparation of targeting vector DNA
4. Preparation of microinjection buffer
5. Preparation of aliquots for pronuclear microinjection
6. Genotyping of blastocysts derived from microinjected zygotes

Plasmid maps

pCAG-Cas9-162A
pU6-sgRosa26-1_CBh-Cas9-T2A-BF

Supplementary Methods

1. Production of sgRNA

1.1. Generating a T7-PCR template for in vitro transcription ●TIMING 1 d

For sgRNA preparation amplify the sgRNA region from the sgRNA plasmid pU6-sgRosa26-1_CBh-Cas9-T2A-BFP (Addgene #64214) using primers T7-sgRosa26-1-for and sgRNA-rev:

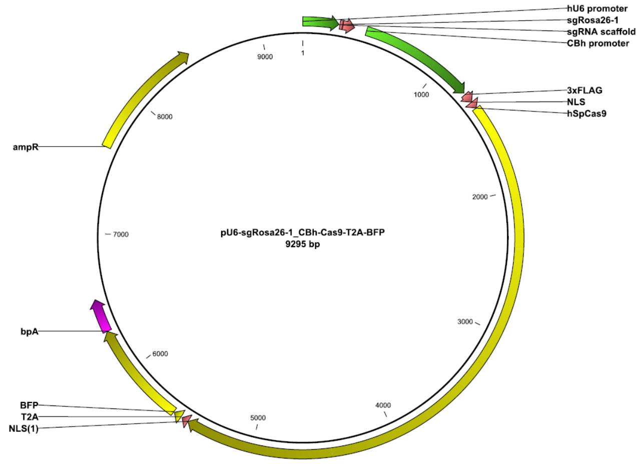

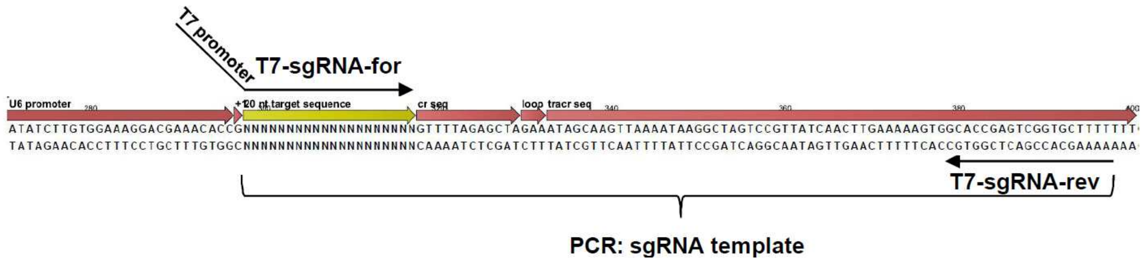

Primer	Sequence (5′–3′)
T7-sgRosa26-for	**TTAATACGACTCACTATAGGACTCCAGTCTTTCTAGAAGAGT**
T7-sgRNA-rev	**AAAAGCACCGACTCGGTGCC**

Using these primers, set up 3-5 x 50ul PCR reactions:

Reagents	50µl PCR reaction	For 5 x 50µl PCR reactions
pU6-sgRosa26-1_CBh-Cas9-T2A-BFP	2 µl	10 µl
10x (5x) buffer	5 (10) µl	25 (50) µl
dNTPs (10 mM)	1 µl	5 µl
Primer T7-sgRosa26-for (50 pMol/µl)	1 µl	5 µl
Primer T7-sgRNA-rev (50 pMol/µl)	1 µl	5 µl
Herculase II	1 ul	5 ul
H_20	39 (34)µl	195 (170) µl
PCR program:<br><br>95°C 3min; 35 cycles of [95°C-20s; 54-58°C-20s; 72°C-20s]; 72°C-3 min		

• Run 5 ul of the PCR product on a 2% agarose gel to estimate its concentration and to verify that the product is unique and has the expected size of 100 bp. PCR should be optimized to yield a single specific band to avoid gel purification.

• Pool the 3-5 PCR reactions for purification using the Qiagen PCR purification kit. Elute with 30 µl RNAse free water. Quantify using a Biophotometer or Nanodrop; a concentration of $\geq$ 125 ng/ul is required for IVT (see below).

***PAUSE POINT** samples can be stored at -20°C for 6 months*

1.2 In vitro Transcription (IVT) ●TIMING 1 d

The sgRNA PCR product is used as template for in-vitro transcription using the Ambion MEGA shortscript T7 Kit (AM1354) following the manual, for a 20 µl IVT reaction add:

- 1 µg of the sgRNA PCR template, in a volume up to 8 µl
- 2 µL 10X Reaction Buffer
- 2 µL ATP Solution (75 mM)
- 2 µL CTP Solution (75 mM)
- 2 µL GTP Solution (75 mM)
- 2 µL UTP Solution (75 mM)
- x µl water (Nuclease-free), to 18 µL final volume
- 2 µL T7 Enzyme Mix
- ➢ Mix, incubate 2-4 h at 37°C

1.3 sgRNA purification

Use the Ambion MEGAclear kit (AM1908) to purify the *in vitro* transcribed RNA following the manual:

To the 20 µl transcription reaction add:

- 80 µl **elution solution**
- 350 µl **binding buffer** (20 ml EtOH added to new bottle)
- 250 µl **Ethanol** (100%)

- Mix and apply to column in the collection tube
- Spin column for 1 min at 12.000 rpm
- Wash column 2x with 500 µl wash solution, spin 1 min, discard flowthrough
- Spin the empty column for 30 sec at 12.000 rpm
- Place column into a new collection tube
- Apply 50 µl elution solution, close the lid, place into an incubator at 65-70°C for 5-10 min (Alternatively, apply 50 ul of elution solution preheated to 95C)
- Spin for 1 min at 12.000 rpm
- Apply another 50 µl of elution solution, close the lid, place into an incubator at 65-70°C for 5-10 min (Alternatively, apply another 50 ul of elution solution preheated to 95C)
- Spin for 1 min at 12.000 rpm, save the eluate, discard the column

To the eluate (100 µl) add:

- 10 µl 5 M Ammoniumacetate (1/10 Vol)
- 275 µl Ethanol (2.5 Vol)

Incubate at -20°C for $\geq$ 30 min or **overnight**

PAUSE POINT *samples can be left overnight at -20°C for precipitation*

- Spin for 15 min at 15.000 g
- Wash the pellet with 500 µl of 70% Ethanol, spin 1 min, discard supernatant
- Spin the dry tube for 10 sec and take off last traces of Ethanol
- Air dry the open tube for 3-5 min

Resuspend the pellet in 30 µl $T_{10}E_{0.1}$ injection buffer, incubate 5-10 min at 37°C, mix

- use 1 ul to measure concentration by OD_{260} (expected yield: 0.5 - 1 µg/ul)
- run 1 ug RNA on a 2% agarose gel (RNAse-free) (sgRNA size: 100 nt)

▲**CRITICAL STEP** Run 500 ng of denatured sgRNA (70°C for 10 min) on a 2% agarose gel to confirm integrity before injection. Smeared bands indicate degradation and these samples should be discarded. Take care to use an RNAse free gel chamber and buffers !

PAUSE POINT *Store the samples at −80 ℃ for the preparation of injection aliquots (up to 2 months).*

2. Production of Cas9-162A mRNA

2.1 Isolation of the T7-Cas9 template for in vitro transcription ●TIMING 1 d

For IVT of Cas9 mRNA a 4.5 kb XbaI-AsiSI fragment including the T7 promoter, Cas9 coding region and a poly A tail (162A; plasmid coded), is isolated from the plasmid pCAG-Cas9-162A. AscI is used in addition to cut the plasmid´s backbone into smaller fragments. Map of pCAG-Cas9-162A:

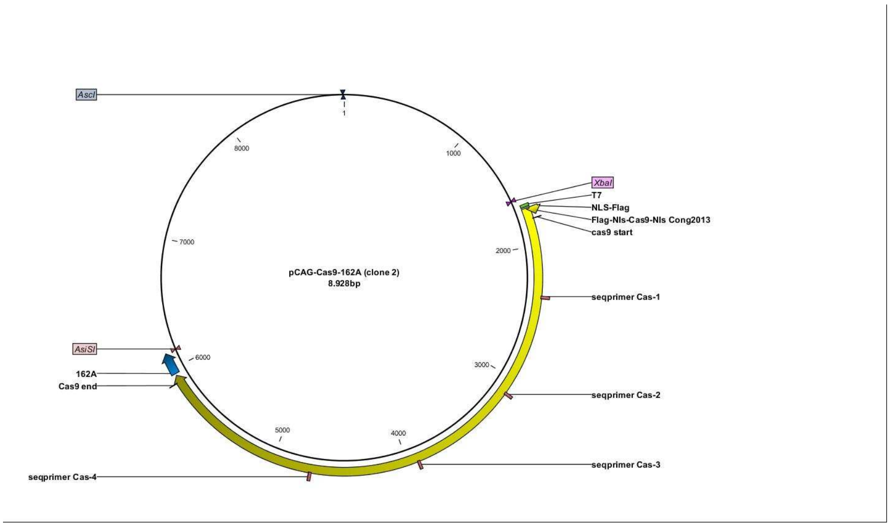

1. Set up a restriction enzyme digestion as follows:

Reagent	In 100 µl
pCAG-Cas9-162pA plasmid DNA	10 µg
10x buffer NEB CutSmart	10 µl
XbaI (20 U/ul, NEB 0145)	2 µl
AsiSI (10 U/ul, NEB R0630)	4 µl
AscI (10 U/ul, NEB R0558)	4 µl
H_20	fill up to 100 µl

2. Incubate at 37°C for 2h

3. Add 20 µl of 6x gel loading buffer to the digestion reaction and load 10 µl into the first lane of a 0.9 % agarose gel (12 x 12 cm), next to a size marker in the second lane. Keep three or more lanes free and load the remaining volume (110 µl) into the next 3-4 lanes. Run the gel at 120V for 1.5h and cut off the left 2 lanes and take an image of this gel segment under short wave UV light. There should be three fragments of 1.6, 2.8 and 4.5 kb visible.

4a. **Option A**: If the digestion looks ok, cut out the largest, 4.5 kb (XbaI-AsiSI) band (representing the T7-Cas9-162A IVT template) under **long wave UV light** (365 nm) and isolate the DNA using the Qiagen gel extraction kit with a final elution volume of 30 µl. (Alternatively, for higher concentrations, use the Qiagen Minelute gel extraction kit (Qiagen #28604) and 10 µl elution buffer)

4b. **Option B**: If the digestion looks ok, precipitate the DNA by adding 10 µl 3 MNaAc + 250 µl EtOH > 30 min/-20C. Spin 10 min, wash w. 70% EtOH, air dry & solve in 25 µl water (nuclease-free)

5. Determine the DNA concentration using a Biophotometer or Nanodrop, each IVT reaction needs 1 µg of the fragment in a volume of $\leq$ 6 ul, therefore a DNA concentration of $\geq$ 166 ng/µl is necessary.

2.2 Cas9 in vitro Transcription (IVT) ●TIMING 1 d

The Cas9-162pA template is in-vitro transcribed using the Ambion mMESSAGE mMACHINE®T7 Ultra Kit (AM 1345) following the manual, for a 20 µl IVT reaction add:

- 1 µg of the template DNA fragment, in a volume up to 6 µl
- 10 µl T7 2x NTP/ARCA
- 2 µl 10x buffer (at room temperature)
- x µl water (Nuclease-free), to a final volume of 18 µl
- 2 µl enzyme mix

➢ Mix, incubate for 2h at 37°C

2.3a Cas9 mRNA purification: Option 1 - MegaClearkit

Use the Ambion MEGAclear kit (AM1908) to purify the *in vitro* transcribed mRNA following the manual:

To the 20 µl transcription reaction add:

- - 80 µl **elution solution**
- - 350 µl **binding buffer** (20 ml EtOH added to new bottle)
- - 250 µl **Ethanol** (100%)
- Mix and apply to column in the collection tube
- Spin column for 1 min at 10-**15.000** g
- Wash column 2x with 500 µl wash solution, spin 1 min, discard flowthrough
- Spin the empty column for 30 sec at 10-15.000 g
- Place column into a new collection tube
- Apply 50 µl elution solution, close the lid, place into an incubator at 65-70°C for 5-10 min (Alternatively, apply 50 ul of elution solution, preheated to 95C)
- Spin for 1 min at 10-15.000 g

- Apply another 50 µl of elution solution, close the lid, place into an incubator at 65-70°C for 5-10 min (Alternatively, apply another 50 ul of elution solution, preheated to 95C)
- Spin for 1 min at 10-15.000 g, save the eluate, discard the column

Or: 2.3b Cas9 mRNA purification: Option 2 - Oligotex kit

Use the Qiagen mRNA mini kit (#70022) to purify the *in vitro* transcribed mRNA following the manual:

To the 20 µl transcription reaction add:

180 µl Water (nuclease-free)
200 µl buffer OBB (if precipitate present, warm at 37C)
20 µl Oligotex suspension (before warmed to 37C and vortexed)

- Mix and incubate at 70 C for 3 min
- place at room temperature for 10 min
- apply to column in the collection tube
- Spin the column for 1 min at 12.000 rpm
- Transfer column into a new collection tube
- Wash column with 400 µl buffer OW2, spin 1 min, discard flowthrough
- Wash column again with 400 µl buffer OW2, spin 1 min
- Transfer column into a new collection tube
- apply 25 µl of elution buffer OEB (preheated to 70C), pipette 3-4x to resuspend resin
- Spin for 1 min at 12.000 rpm
- Apply another 25 µl of buffer OEB (preheated to 70 C), pipette 3-4x to resuspend resin
- Spin for 1 min at 12.000 rpm, save the eluate, discard the column

2.4 Precipitation

To the eluate (100 µl) add:

- 5 µl 5 M Ammoniumacetate (1/10 Vol)
- 138 µl Ethanol (2.5 Vol)

Incubate at -20°C for ≥ 30 min or **overnight**

PAUSE POINT *samples can be left overnight at -20°C for precipitation*

- Spin for 15 min at 15.000 g
- Wash the pellet with 500 µl of 70% Ethanol, spin 1 min, discard supernatant
- Spin the dry tube for 10 sec and take off last traces of Ethanol
- Dry the pellet at air in the tube with open lid for 3-5 min

Resuspend the pellet in 30 µl $T_{10}E_{0.1}$ injection buffer, incubate 5-10 min at 37°C, mix

PAUSE POINT *Store the samples at −80 ℃ for the preparation of injection aliquots (up to 2 months).*

2.5 RNA analysis

- use 1 µl to measure concentration by OD_{260} (expected yield: 0.5 - 1 µg/ul)

Gel option A: run 1 µg RNA on a standard 0.9 % agarose gel (RNAse-free) (mRNA size: 4411 nt). Run 1 µg of heat denatured mRNA (70^{0}C for 10 min) on a 0.9% agarose gel (TAE/TBE) (1 µg/ml Ethidiumbromide) to confirm integrity before injection. Smeared bands indicate degradation and these samples should be discarded. Take care to use an RNAse free gel chamber and buffers !

Gel option B: For optimal results use denaturing (glyoxal) gel electrophoresis and Northern Max-Gly reagents from Ambion: Mix 1 ug RNA in 10 µl water (nuclease-free) with 10 ul NorthernMax®-Gly Sample Loading Dye (AM8551), incubate at 50C for 30 min. Load on a 0.9% agarose gel prepared with NorthernMax®-Gly 10x gel prep/running buffer (AM8678) and 1 µg/ml Ethidiumbromide. As size marker load 2 µg of RNA Millenium size marker (AM7150), treated as above with AM8551.

Example gel:

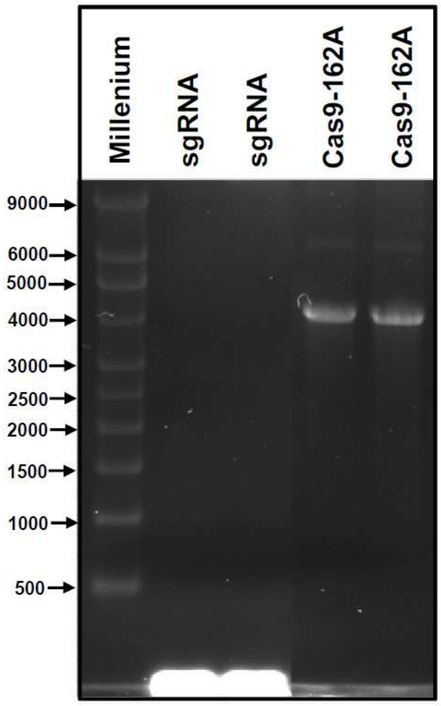

Gel option C: Run the RNA in the Agilent Bioanalyser using the Agilent RNA 6000 Nano kit (#5067-1511), following Agilents instruction manual: http://www.chem.agilent.com/Library/usermanuals/Public/G2938-90034_RNA6000Nano_KG.pdf

3. Preparation of targeting vector DNA ●TIMING 2h

1. Precipitate 15 µg of plasmid DNA (Qiagen Maxiprep) of the gene targeting vector by adding 0.1 volumes of 3 M sodium acetate and 2.5 volumes of ethanol. Incubate the mixture for 30 min at RT and pellet the DNA by centrifugation at 16.000*g* for 10 min at RT.

2. Discard the supernatant and wash the pellet by the addition of 500 ul of 70% (vol/vol) ethanol (prepared with embryo-tested water, available from the TCF). Centrifuge the tube at

16.000*g* for 2 min at RT, discard the supernatant and repeat the washing step. Discard the supernatant and air-dry the pellet for 5 min.

3. Resuspend the DNA pellet in 30 µl of $T_{10}E_{0.1}$ microinjection buffer for 30 min at 37 ℃. Mix by pipetting and use 1 µl to determine the DNA concentration using a Biophotometer or Nanodrop. Store the DNA solution at −80 ℃ for the preparation of microinjection samples.

4. Preparation of microinjection buffer ($T_{10}E_{0.1}$)

$T_{10}E_{0.1}$ microinjection buffer contains 10 mM Tris and 0.1 mM EDTA (pH 7.4), made of reagents of the highest purity to avoid embryo toxicity.

Stock preparation:

- 1 M Tris-base stock solution is prepared by dissolving 3030 mg of Trizma base (Sigma ultra T6791, Mw=121,14) in 25 ml of embryo-tested water (Sigma W1503)

- 1 M Tris-acid stock solution is prepared by dissolving 3940 mg of Trizma hydrochloride (Sigma ultra T6666, Mw=157,6) in 25 ml of embryo-tested water.

- 5 mM EDTA stock solution is prepared by dissolving 93 mg of EDTA disodium salt dihydrate (Sigma E4884, Mw=372,24) in 50 ml embryo tested water.

For preparation of 25 ml $T_{10}E_{0.1}$ buffer combine in a 50 ml Falcon tube:

- 24.25 ml embryo-tested water
- 40.85 µl Tris-base stock (1 M)
- 209 µl TRIS-acid stock (1 M)
- 500 µl EDTA stock (5 mM)

Give this mixture into a 20-ml disposable syringe and filter through a Millex GV filter (Millipore, cat. no. SLGV033RS), discarding the first 5 ml of the filtrate. Use 100ul buffer to confirm that the pH value falls into the range of 7.0–7.5, by using pH test strips. Store $T_{10}E_{0.1}$ injection buffer in 1 ml aliquots at −80 ℃ for up to 2 years.

5. Preparation of aliquots for pronuclear microinjection ●TIMING 2 h

Thaw the sgRNA and Cas9 mRNA the DNA and a tube of $T_{10}E_{0.1}$ microinjection buffer. For each day of microinjection, a single-use aliquot of 30 µl of injection solution is used, in total a volume of 150 µl injection solution is needed for a complete pronucleus microinjection experiment of up to 5 microinjection days (5 x 10 superovulated female mice for zygote production). Standard concentrations are:

Cas9 mRNA:	**25 ng/µl**
sgRNA:	**12.5 ng/µl**
Targeting vector DNA:	**20 ng/µl**

To prepare 150 µl of a master mix, combine in a clean, dust-free 1.5-ml tube:

- 3750 ng of Cas9 mRNA
- 1875 ng of sgRNA (each)
- 3000 ng targeting vector
- adjust with $T_{10}E_{0.1}$ buffer to a final volume of 150 ul

For the removal of dust particles, the master mix is filtered by using a centrifugal filter (Ultrafree, PFTE, Millipore, cat. no. UFC30LG25, available from the TCF):

- ➢ load the master mix into the filter cartridge
- ➢ centrifuge at 12,000*g* for 1 min at RT.
- ➢ Pipette the filtrate in aliquots of 30 µl into five clean 1.5-ml tubes, labelled with the TCF project number and date of preparation.

Store the microinjection samples at −80 ℃ and them over to the TCF microinjection service. One tube is used for each day of embryo injection. Microinjection samples can be safely used for embryo injections up to 6 (-8) weeks after RNA production.

▲CRITICAL STEP Avoid any contamination of the microinjection samples with dust particles, which will block injection capillaries, by using 1.5-ml tubes taken from a freshly opened bag. Best use individually packed Eppendorf 1.5 ml \`Biopure´ tubes (Cat No.: 0030 121.589).

6. Genotyping of blastocysts derived from microinjected zygotes

6.1 Blastocyst collection

Add 10 µl Quick DNA extraction solution (QuickExtract™ DNA Extraction Solution, QE09050, Epicentre) into each well of a 96 well PCR plate. A single blastocyst is mouth-pipetted into each well.

6.2. DNA Denaturation

PCR plates are briefly centrifuged and proceeded for DNA denaturation

68^0C	15 min
95^0C	15 min

6.3 1st PCR

performed nested PCR reaction. Use Herculase II Fusion DNA Polymerase.

Nested PCR for Rosa26 locus	R26F1	CCAAAGTCGCTCTGAGTTGTTATCAGT
	R26R1	GGAGCGGGAGAAATGGATATGAAG

1st PCR	In 25µl PCR
Denatured DNA	3 (Blastocyst) or 5 µl (Morula)
5x buffer	5
dNTP	0.25
Primer For	0.5
Primer Rev	0.5
DNA polymerase	0.3
H_20	Up to 25

98°C 3min; 35 cycles of [95°C-20s; 60°C-20s; 72°C-20s]; 72°C-3 min

6.4 2nd PCR

Nested PCR for Rosa26 locus	R26F2<br>R26R2	GCCTCCTGGCTTCTGAGGACCG<br>TCTGTGGGAAGTCTTGTCCCTCC

2nd PCR	In 25µl PCR
1st PCR product	2
5x buffer	5
dNTP	0.25
Primer For	0.5
Primer Rev	0.5
DNA polymerase	0.3
H_20	Up to 25

98°C 3min; 35 cycles of [95°C-20s; 60°C-20s; 72°C-20s]; 72°C-3 min

PCR products are purified using PCR extraction kit

Product = **196 bp** (XbaI digested: 115 + 85 bp)

III. Plasmid maps

Plasmid pCAG-Cas9-162A

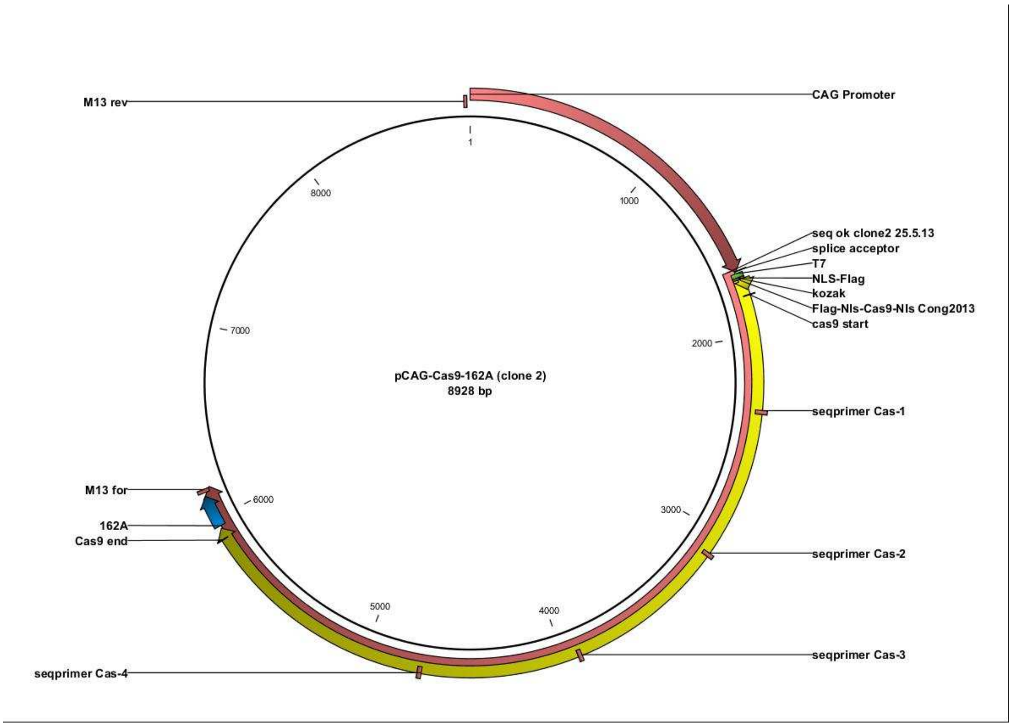


```
LOCUS        pCAG-Cas9-162A_(clone_2)      8928 bp     DNA      circular UNA
18-APR-2013
ACCESSION   pCAG-cre-bpA
COMMENT     This file is created by Vector NTI
            http://www.invitrogen.com/
FEATURES             Location/Qualifiers
     misc_feature    1..1671
                     /vntifkey="21"
                     /label="CAG Promoter"
                     /note="Oriinal location: 87..1753"
     misc_feature    1660..6162
                     /label="seq ok clone2 25.5.13"
     misc_feature    1670..1671
                     /vntifkey="21"
                     /label="splice acceptor"
                     /note="Oriinal location: 1752..1753"
     misc_feature    1681..5949
                     /note="originates from pCAG-Cas9-bpA_PacI_MluI"
                     /label=Insert
     misc_feature    1681..5949
                     /note="originates     from     pCAG-Cas9-96A     (clone
15)_PacI_MluI"
                     /label=Insert
     promoter        1681..1703
                     /label=T7
     Region          1704..1712
                     /label=kozak
     misc_feature    1704..1712
                     /label=kozak
     CDS             1713..5948
                     /label="Flag-Nls-Cas9-Nls Cong2013"
     CDS             1713..1772
                     /label=NLS-Flag
     CDS             1797..1799
                     /label="cas9 start"
```

```
     primer          2366..2385
                     /label="seqprimer Cas-1"
     primer          3109..3128
                     /label="seqprimer Cas-2"
     primer          3908..3927
                     /label="seqprimer Cas-3"
     primer          4704..4724
                     /label="seqprimer Cas-4"
     CDS             5895..5897
                     /label="Cas9 end"
     polyA_site      5962..6123
                     /vntifkey="26"
                     /label=163-166A
                     /label=162A
     misc_feature    complement(6143..6159)
                     /vntifkey="21"
                     /label="M13 for"
     misc_feature    8898..8913
                     /vntifkey="21"
                     /label="M13 rev"
ORIGIN
        1 GACATTGATT ATTGACTAGT TATTAATAGT AATCAATTAC GGGGTCATTA GTTCATAGCC
       61 CATATATGGA GTTCCGCGTT ACATAACTTA CGGTAAATGG CCCGCCTGGC TGACCGCCCA
      121 ACGACCCCCG CCCATTGACG TCAATAATGA CGTATGTTCC CATAGTAACG CCAATAGGGA
      181 CTTTCCATTG ACGTCAATGG GTGGAGTATT TACGGTAAAC TGCCCACTTG GCAGTACATC
      241 AAGTGTATCA TATGCCAAGT ACGCCCCCTA TTGACGTCAA TGACGGTAAA TGGCCCGCCT
      301 GGCATTATGC CCAGTACATG ACCTTATGGG ACTTTCCTAC TTGGCAGTAC ATCTACGTAT
      361 TAGTCATCGC TATTACCATG GTCGAGGTGA GCCCCACGTT CTGCTTCACT CTCCCCATCT
      421 CCCCCCCCTC CCCACCCCCA ATTTTGTATT TATTTATTTT TTAATTATTT TGTGCAGCGA
      481 TGGGGGCGGG GGGGGGGGGG GGGCGCGCGC CAGGCGGGGC GGGGCGGGGC GAGGGGCGGG
      541 GCGGGGCGAG GCGGAGAGGT GCGGCGGCAG CCAATCAGAG CGGCGCGCTC CGAAAGTTTC
      601 CTTTTATGGC GAGGCGGCGG CGGCGGCGGC CCTATAAAAA GCGAAGCGCG CGGCGGGCGG
      661 GAGTCGCTGC GTCGCGCCTT CGCCCCGTGC CCCGCTCCGC CGCCGCCTCG CGCCGCCCGC
      721 CCCGGCTCTG ACTGACCGCG TTACTCCCAC AGGTGAGCGG GCGGGACGGC CCTTCTCCTC
      781 CGGGCTGTAA TTAGCGCTTG GTTTAATGAC GGCTCGTTTC TTTTCTGTGG CTGCGTGAAA
      841 GCCTTAAAGG GCTCCGGGAG GGCCCTTTGT GCGGGGGGGA GCGGCTCGGG GGGTGCGTGC
      901 GTGTGTGTGT GCGTGGGGAG CGCCGCGTGC GGCCCGCGCT GCCCGGCGGC TGTGAGCGCT
      961 GCGGGCGCGG CGCGGGGCTT TGTGCGCTCC GCGTGTGCGC GAGGGGAGCG CGGCCGGGGG
     1021 CGGTGCCCCG CGGTGCGGGG GGGCTGCGAG GGGAACAAAG GCTGCGTGCG GGGTGTGTGC
     1081 GTGGGGGGGT GAGCAGGGGG TGTGGGCGCG GCGGTCGGGC TGTAACCCCC CCCTGCACCC
     1141 CCCTCCCCGA GTTGCTGAGC ACGGCCCGGC TTCGGGTGCG GGGCTCCGTG CGGGGCGTGG
     1201 CGCGGGGCTC GCCGTGCCGG GCGGGGGGTG GCGGCAGGTG GGGGTGCCGG GCGGGGCGGG
     1261 GCCGCCTCGG GCCGGGGAGG GCTCGGGGGA GGGGCGCGGC GGCCCCGGAG CGCCGGCGGC
     1321 TGTCGAGGCG CGGCGAGCCG CAGCCATTGC CTTTTATGGT AATCGTGCGA GAGGGCGCAG
     1381 GGACTTCCTT TGTCCCAAAT CTGGCGGAGC CGAAATCTGG GAGGCGCCGC CGCACCCCCT
     1441 CTAGCGGGCG CGGGCGAAGC GGTGCGGCGC CGGCAGGAAG GAAATGGGCG GGGAGGGCCT
     1501 TCGTGCGTCG CCGCGCCGCC GTCCCCTTCT CCATCTCCAG CCTCGGGGCT GCCGCAGGGG
     1561 GACGGCTGCC TTCGGGGGGG ACGGGGCAGG GCGGGGTTCG GCTTCTGGCG TGTGACCGGC
     1621 GGCTCTAGAG CCTCTGCTAA CCATGTTCAT GCCTTCTTCT TTTTCCTACA GATCCTTAAT
     1681 TAATAATACG ACTCACTATA GGGgccgcca ccATGGGACC TAAGAAAAAG AGGAAGGTGG
     1741 CGGCCGCTGA CTACAAGGAT GACGACGATA AAGGTATCCA CGGAGTCCCA GCAGCCGACA
     1801 AGAAGTACAG CATCGGCCTG GACATCGGCA CCAACTCTGT GGGCTGGGCC GTGATCACCG
     1861 ACGAGTACAA GGTGCCCAGC AAGAAATTCA AGGTGCTGGG CAACACCGAC CGGCACAGCA
     1921 TCAAGAAGAA CCTGATCGGA GCCCTGCTGT TCGACAGCGG CGAAACAGCC GAGGCCACCC
     1981 GGCTGAAGAG AACCGCCAGA AGAAGATACA CCAGACGGAA GAACCGGATC TGCTATCTGC
     2041 AAGAGATCTT CAGCAACGAG ATGGCCAAGG TGGACGACAG CTTCTTCCAC AGACTGGAAG
     2101 AGTCCTTCCT GGTGGAAGAG GATAAGAAGC ACGAGCGGCA CCCCATCTTC GGCAACATCG
     2161 TGGACGAGGT GGCCTACCAC GAGAAGTACC CCACCATCTA CCACCTGAGA AAGAAACTGG
     2221 TGGACAGCAC CGACAAGGCC GACCTGCGGC TGATCTATCT GGCCCTGGCC CACATGATCA
     2281 AGTTCCGGGG CCACTTCCTG ATCGAGGGCG ACCTGAACCC CGACAACAGC GACGTGGACA
     2341 AGCTGTTCAT CCAGCTGGTG CAGACCTACA ACCAGCTGTT CGAGGAAAAC CCCATCAACG
     2401 CCAGCGGCGT GGACGCCAAG GCCATCCTGT CTGCCAGACT GAGCAAGAGC AGACGGCTGG
     2461 AAAATCTGAT CGCCCAGCTG CCCGGCGAGA AGAAGAATGG CCTGTTCGGC AACCTGATTG
     2521 CCCTGAGCCT GGGCCTGACC CCCAACTTCA AGAGCAACTT CGACCTGGCC GAGGATGCCA
     2581 AACTGCAGCT GAGCAAGGAC ACCTACGACG ACGACCTGGA CAACCTGCTG GCCCAGATCG
     2641 GCGACCAGTA CGCCGACCTG TTTCTGGCCG CCAAGAACCT GTCCGACGCC ATCCTGCTGA
     2701 GCGACATCCT GAGAGTGAAC ACCGAGATCA CCAAGGCCCC CCTGAGCGCC TCTATGATCA
     2761 AGAGATACGA CGAGCACCAC CAGGACCTGA CCCTGCTGAA AGCTCTCGTG CGGCAGCAGC
     2821 TGCCTGAGAA GTACAAAGAG ATTTTCTTCG ACCAGAGCAA GAACGGCTAC GCCGGCTACA
     2881 TTGACGGCGG AGCCAGCCAG GAAGAGTTCT ACAAGTTCAT CAAGCCCATC CTGGAAAAGA
```

2941 TGGACGGCAC CGAGGAACTG CTCGTGAAGC TGAACAGAGA GGACCTGCTG CGGAAGCAGC
3001 GGACCTTCGA CAACGGCAGC ATCCCCCACC AGATCCACCT GGGAGAGCTG CACGCCATTC
3061 TGCGGCGGCA GGAAGATTTT TACCCATTCC TGAAGGACAA CCGGGAAAAG ATCGAGAAGA
3121 TCCTGACCTT CCGCATCCCC TACTACGTGG GCCCTCTGGC CAGGGGAAAC AGCAGATTCG
3181 CCTGGATGAC CAGAAAGAGC GAGGAAACCA TCACCCCCTG GAACTTCGAG GAAGTGGTGG
3241 ACAAGGGCGC TTCCGCCCAG AGCTTCATCG AGCGGATGAC CAACTTCGAT AAGAACCTGC
3301 CCAACGAGAA GGTGCTGCCC AAGCACAGCC TGCTGTACGA GTACTTCACC GTGTATAACG
3361 AGCTGACCAA AGTGAAATAC GTGACCGAGG GAATGAGAAA GCCCGCCTTC CTGAGCGGCG
3421 AGCAGAAAAA GGCCATCGTG GACCTGCTGT TCAAGACCAA CCGGAAAGTG ACCGTGAAGC
3481 AGCTGAAAGA GGACTACTTC AAGAAAATCG AGTGCTTCGA CTCCGTGGAA ATCTCCGGCG
3541 TGGAAGATCG GTTCAACGCC TCCCTGGGCA CATACCACGA TCTGCTGAAA ATTATCAAGG
3601 ACAAGGACTT CCTGGACAAT GAGGAAAACG AGGACATTCT GGAAGATATC GTGCTGACCC
3661 TGACACTGTT TGAGGACAGA GAGATGATCG AGGAACGGCT GAAAACCTAT GCCCACCTGT
3721 TCGACGACAA AGTGATGAAG CAGCTGAAGC GGCGGAGATA CACCGGCTGG GGCAGGCTGA
3781 GCCGGAAGCT GATCAACGGC ATCCGGGACA AGCAGTCCGG CAAGACAATC CTGGATTTCC
3841 TGAAGTCCGA CGGCTTCGCC AACAGAAACT TCATGCAGCT GATCCACGAC GACAGCCTGA
3901 CCTTTAAAGA GGACATCCAG AAAGCCCAGG TGTCCGGCCA GGGCGATAGC CTGCACGAGC
3961 ACATTGCCAA TCTGGCCGGC AGCCCCGCCA TTAAGAAGGG CATCCTGCAG ACAGTGAAGG
4021 TGGTGGACGA GCTCGTGAAA GTGATGGGCC GGCACAAGCC CGAGAACATC GTGATCGAAA
4081 TGGCCAGAGA GAACCAGACC ACCCAGAAGG GACAGAAGAA CAGCCGCGAG AGAATGAAGC
4141 GGATCGAAGA GGGCATCAAA GAGCTGGGCA GCCAGATCCT GAAAGAACAC CCCGTGGAAA
4201 ACACCCAGCT GCAGAACGAG AAGCTGTACC TGTACTACCT GCAGAATGGG CGGGATATGT
4261 ACGTGGACCA GGAACTGGAC ATCAACCGGC TGTCCGACTA CGATGTGGAC CATATCGTGC
4321 CTCAGAGCTT TCTGAAGGAC GACTCCATCG ACAACAAGGT GCTGACCAGA AGCGACAAGA
4381 ACCGGGGCAA GAGCGACAAC GTGCCCTCCG AAGAGGTCGT GAAGAAGATG AAGAACTACT
4441 GGCGGCAGCT GCTGAACGCC AAGCTGATTA CCCAGAGAAA GTTCGACAAT CTGACCAAGG
4501 CCGAGAGAGG CGGCCTGAGC GAACTGGATA AGGCCGGCTT CATCAAGAGA CAGCTGGTGG
4561 AAACCCGGCA GATCACAAAG CACGTGGCAC AGATCCTGGA CTCCCGGATG AACACTAAGT
4621 ACGACGAGAA TGACAAGCTG ATCCGGGAAG TGAAAGTGAT CACCCTGAAG TCCAAGCTGG
4681 TGTCCGATTT CCGGAAGGAT TTCCAGTTTT ACAAAGTGCG CGAGATCAAC AACTACCACC
4741 ACGCCCACGA CGCCTACCTG AACGCCGTCG TGGGAACCGC CCTGATCAAA AAGTACCCTA
4801 AGCTGGAAAG CGAGTTCGTG TACGGCGACT ACAAGGTGTA CGACGTGCGG AAGATGATCG
4861 CCAAGAGCGA GCAGGAAATC GGCAAGGCTA CCGCCAAGTA CTTCTTCTAC AGCAACATCA
4921 TGAACTTTTT CAAGACCGAG ATTACCCTGG CCAACGGCGA GATCCGGAAG CGGCCTCTGA
4981 TCGAGACAAA CGGCGAAACC GGGGAGATCG TGTGGGATAA GGGCCGGGAT TTTGCCACCG
5041 TGCGGAAAGT GCTGAGCATG CCCCAAGTGA ATATCGTGAA AAAGACCGAG GTGCAGACAG
5101 GCGGCTTCAG CAAAGAGTCT ATCCTGCCCA AGAGGAACAG CGATAAGCTG ATCGCCAGAA
5161 AGAAGGACTG GGACCCTAAG AAGTACGGCG GCTTCGACAG CCCCACCGTG GCCTATTCTG
5221 TGCTGGTGGT GGCCAAAGTG GAAAAGGGCA AGTCCAAGAA ACTGAAGAGT GTGAAAGAGC
5281 TGCTGGGGAT CACCATCATG GAAAGAAGCA GCTTCGAGAA GAATCCCATC GACTTTCTGG
5341 AAGCCAAGGG CTACAAAGAA GTGAAAAAGG ACCTGATCAT CAAGCTGCCT AAGTACTCCC
5401 TGTTCGAGCT GGAAAACGGC CGGAAGAGAA TGCTGGCCTC TGCCGGCGAA CTGCAGAAGG
5461 GAAACGAACT GGCCCTGCCC TCCAAATATG TGAACTTCCT GTACCTGGCC AGCCACTATG
5521 AGAAGCTGAA GGGCTCCCCC GAGGATAATG AGCAGAAACA GCTGTTTGTG GAACAGCACA
5581 AGCACTACCT GGACGAGATC ATCGAGCAGA TCAGCGAGTT CTCCAAGAGA GTGATCCTGG
5641 CCGACGCTAA TCTGGACAAA GTGCTGTCCG CCTACAACAA GCACCGGGAT AAGCCCATCA
5701 GAGAGCAGGC CGAGAATATC ATCCACCTGT TTACCCTGAC CAATCTGGGA GCCCCTGCCG
5761 CCTTCAAGTA CTTTGACACC ACCATCGACC GGAAGAGGTA CACCAGCACC AAAGAGGTGC
5821 TGGACGCCAC CCTGATCCAC CAGAGCATCA CCGGCCTGTA CGAGACACGG ATCGACCTGT
5881 CTCAGCTGGG AGGCGACAAG CGTCCTGCTG CTACTAAGAA AGCTGGTCAA GCTAAGAAAA
5941 AGAAATGAAC GCGTCTTAAA TAAAAAAAAA AAAAAAAAAA AAAAAAAAAA AAAAAAAAAA
6001 AAAAAAAAAA AAAAAAAAAA AAAAAAAAAA AAAAAAAAAA AAAAAAAAAA AAAAAAAAAA
6061 AAAAAAAAAA AAAAAAAAAA AAAAAAAAAA AAAAAAAAAA AAAAAAAAAA AAAAAAAAAA
6121 AAAGCGATCG CGGGCGGCCG CCACTGGCCG TCGTTTTACA ACGTCGTGAC TGGGAAAACC
6181 CTGGCGTTAC CCAACTTAAT CGCCTTGCAG CACATCCCCC TTTCGCCAGC TGGCGTAATA
6241 GCGAAGAGGC CCGCACCGAT CGCCCTTCCC AACAGTTGCG CAGCCTGAAT GGCGAATGGG
6301 ACGCGCCCTG TAGCGGCGCA TTAAGCGCGG CGGGTGTGGT GGTTACGCGC AGCGTGACCG
6361 CTACACTTGC CAGCGCCCTA GCGCCCGCTC CTTTCGCTTT CTTCCCTTCC TTTCTCGCCA
6421 CGTTCGCCGG CTTTCCCCGT CAAGCTCTAA ATCGGGGGCT CCCTTTAGGG TTCCGATTTA
6481 GTGCTTTACG GCACCTCGAC CCCAAAAAAC TTGATTAGGG TGATGGTTCA CGTAGTGGGC
6541 CATCGCCCTG ATAGACGGTT TTTCGCCCTT TGACGTTGGA GTCCACGTTC TTTAATAGTG
6601 GACTCTTGTT CCAAACTGGA ACAACACTCA ACCCTATCTC GGTCTATTCT TTTGATTTAT
6661 AAGGGATTTT GCCGATTTCG GCCTATTGGT TAAAAAATGA GCTGATTTAA CAAAAATTTA
6721 ACGCGAATTT TAACAAAATA TTAACGCTTA CAATTTAGGT GGCACTTTTC GGGGAAATGT
6781 GCGCGGAACC CCTATTTGTT TATTTTTCTA AATACATTCA AATATGTATC CGCTCATGAG
6841 ACAATAACCC TGATAAATGC TTCAATAATA TTGAAAAAGG AAGAGTATGA GTATTCAACA
6901 TTTCCGTGTC GCCCTTATTC CCTTTTTTGC GGCATTTTGC CTTCCTGTTT TTGCTCACCC
6961 AGAAACGCTG GTGAAAGTAA AAGATGCTGA AGATCAGTTG GGTGCACGAG TGGGTTACAT
7021 CGAACTGGAT CTCAACAGCG GTAAGATCCT TGAGAGTTTT CGCCCCGAAG AACGTTTTCC
7081 AATGATGAGC ACTTTTAAAG TTCTGCTATG TGGCGCGGTA TTATCCCGTA TTGACGCCGG

```
7141 GCAAGAGCAA CTCGGTCGCC GCATACACTA TTCTCAGAAT GACTTGGTTG AGTACTCACC
7201 AGTCACAGAA AAGCATCTTA CGGATGGCAT GACAGTAAGA GAATTATGCA GTGCTGCCAT
7261 AACCATGAGT GATAACACTG CGGCCAACTT ACTTCTGACA ACGATCGGAG GACCGAAGGA
7321 GCTAACCGCT TTTTTGCACA ACATGGGGGA TCATGTAACT CGCCTTGATC GTTGGGAACC
7381 GGAGCTGAAT GAAGCCATAC CAAACGACGA GCGTGACACC ACGATGCCTG TAGCAATGGC
7441 AACAACGTTG CGCAAACTAT TAACTGGCGA ACTACTTACT CTAGCTTCCC GGCAACAATT
7501 AATAGACTGG ATGGAGGCGG ATAAAGTTGC AGGACCACTT CTGCGCTCGG CCCTTCCGGC
7561 TGGCTGGTTT ATTGCTGATA AATCTGGAGC CGGTGAGCGT GGGTCTCGCG GTATCATTGC
7621 AGCACTGGGG CCAGATGGTA AGCCCTCCCG TATCGTAGTT ATCTACACGA CGGGGAGTCA
7681 GGCAACTATG GATGAACGAA ATAGACAGAT CGCTGAGATA GGTGCCTCAC TGATTAAGCA
7741 TTGGTAACTG TCAGACCAAG TTTACTCATA TATACTTTAG ATTGATTTAA AACTTCATTT
7801 TTAATTTAAA AGGATCTAGG TGAAGATCCT TTTTGATAAT CTCATGACCA AAATCCCTTA
7861 ACGTGAGTTT TCGTTCCACT GAGCGTCAGA CCCCGTAGAA AAGATCAAAG GATCTTCTTG
7921 AGATCCTTTT TTTCTGCGCG TAATCTGCTG CTTGCAAACA AAAAAACCAC CCGCTACCAG
7981 CGGTGGTTTG TTTGCCGGAT CAAGAGCTAC CAACTCTTTT TCCGAAGGTA ACTGGCTTCA
8041 GCAGAGCGCA GATACCAAAT ACTGTTCTTC TAGTGTAGCC GTAGTTAGGC CACCACTTCA
8101 AGAACTCTGT AGCACCGCCT ACATACCTCG CTCTGCTAAT CCTGTTACCA GTGGCTGCTG
8161 CCAGTGGCGA TAAGTCGTGT CTTACCGGGT TGGACTCAAG ACGATAGTTA CCGGATAAGG
8221 CGCAGCGGTC GGGCTGAACG GGGGGTTCGT GCACACAGCC CAGCTTGGAG CGAACGACCT
8281 ACACCGAACT GAGATACCTA CAGCGTGAGC TATGAGAAAG CGCCACGCTT CCCGAAGGGA
8341 GAAAGGCGGA CAGGTATCCG GTAAGCGGCA GGGTCGGAAC AGGAGAGCGC ACGAGGGAGC
8401 TTCCAGGGGG AAACGCCTGG TATCTTTATA GTCCTGTCGG GTTTCGCCAC CTCTGACTTG
8461 AGCGTCGATT TTTGTGATGC TCGTCAGGGG GGCGGAGCCT ATGGAAAAAC GCCAGCAACG
8521 CGGCCTTTTT ACGGTTCCTG GCCTTTTGCT GGCCTTTTGC TCACATGTTC TTTCCTGCGT
8581 TATCCCCTGA TTCTGTGGAT AACCGTATTA CCGCCTTTGA GTGAGCTGAT ACCGCTCGCC
8641 GCAGCCGAAC GACCGAGCGC AGCGAGTCAG TGAGCGAGGA AGCGGAAGAG CGCCCAATAC
8701 GCAAACCGCC TCTCCCCGCG CGTTGGCCGA TTCATTAATG CAGCTGGCAC GACAGGTTTC
8761 CCGACTGGAA AGCGGGCAGT GAGCGCAACG CAATTAATGT GAGTTAGCTC ACTCATTAGG
8821 CACCCCAGGC TTTACACTTT ATGCTTCCGG CTCGTATGTT GTGTGGAATT GTGAGCGGAT
8881 AACAATTTCA CACAGGAAAC AGCTATGACC ATGAGGCGCG CCGGATTC
```
//

Plasmid pU6-sgRosa26-1_CBh-Cas9-T2A-BFP (Addgene #64214)

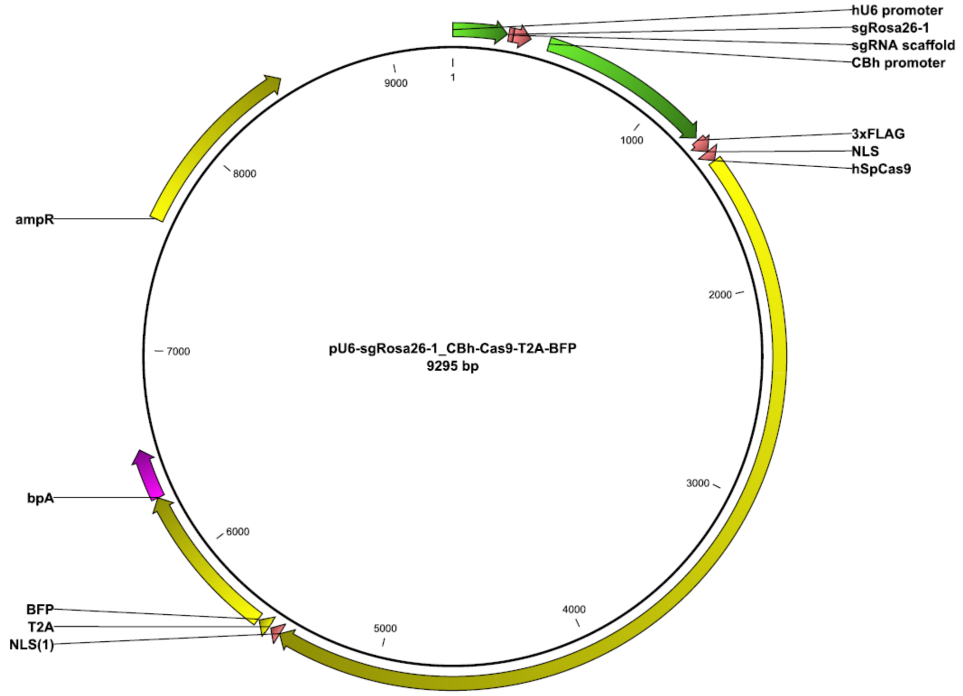


```
LOCUS       pU6-sgRosa26-1_CBh-Cas9-T2A-BFP      9295 bp    DNA
circular UNA 09-MAY-2014
DEFINITION  .
ACCESSION
VERSION
SOURCE      . (unknown)
  ORGANISM  .
            .
COMMENT
FEATURES             Location/Qualifiers
     promoter        1..249
                     /label=hU6
                     /label="hU6 promoter"
     misc_feature    256..276
                     /label=sgRosa26-1
     misc_feature    277..358
                     /label="Guide RNA"
                     /label="sgRNA scaffold"
     promoter        443..1241
                     /label=CBh
                     /label="CBh promoter"
     misc_feature    1254..1322
                     /label=3xFLAG
     misc_feature    1323..1373
                     /label=NLS
     CDS             1374..5474
                     /label=hSpCas9
     misc_feature    5475..5522
                     /label=NLS(1)
     CDS             5532..5586
                     /label=T2A
     CDS             5592..6311
```

```
                     /label=BFP
     polyA_signal    6312..6543
                     /label=pGHpA
                     /label=bpA
     CDS             7615..8475
                     /label=ampR
ORIGIN
        1 CCCCACGAGG GCCTATTTCC CATGATTCCT TCATATTTGC ATATACGATA CAAGGCTGTT
       61 AGAGAGATAA TTGGAATTAA TTTGACTGTA AACACAAAGA TATTAGTACA AAATACGTGA
      121 CGTAGAAAGT AATAATTTCT TGGGTAGTTT GCAGTTTTAA AATTATGTTT TAAAATGGAC
      181 TATCATATGC TTACCGTAAC TTGAAAGTAT TTCGATTTCT TGGCTTTATA TATCTTGTGG
      241 AAAGGACGAA ACACCGACTC CAGTCTTTCT AGAAGAGTTT TAGAGCTAGA AATAGCAAGT
      301 TAAAATAAGG CTAGTCCGTT ATCAACTTGA AAAAGTGGCA CCGAGTCGGT GCTTTTTTGT
      361 TTTAGAGCTA GAAATAGCAA GTTAAAATAA GGCTAGTCCG TTTTTAGCGC GTGCGCCAAT
      421 TCTGCAGACA AATGGCTCTA GAGGTACCCG TTACATAACT TACGGTAAAT GGCCCGCCTG
      481 GCTGACCGCC CAACGACCCC CGCCCATTGA CGTCAATAGT AACGCCAATA GGGACTTTCC
      541 ATTGACGTCA ATGGGTGGAG TATTTACGGT AAACTGCCCA CTTGGCAGTA CATCAAGTGT
      601 ATCATATGCC AAGTACGCCC CCTATTGACG TCAATGACGG TAAATGGCCC GCCTGGCATT
      661 GTGCCCAGTA CATGACCTTA TGGGACTTTC CTACTTGGCA GTACATCTAC GTATTAGTCA
      721 TCGCTATTAC CATGGTCGAG GTGAGCCCCA CGTTCTGCTT CACTCTCCCC ATCTCCCCCC
      781 CCTCCCCACC CCCAATTTTG TATTTATTTA TTTTTTAATT ATTTTGTGCA GCGATGGGGG
      841 CGGGGGGGGG GGGGGGGCGC GCGCCAGGCG GGGCGGGGCG GGGCGAGGGG CGGGGCGGGG
      901 CGAGGCGGAG AGGTGCGGCG GCAGCCAATC AGAGCGGCGC GCTCCGAAAG TTTCCTTTTA
      961 TGGCGAGGCG GCGGCGGCGG CGGCCCTATA AAAAGCGAAG CGCGCGGCGG GCGGGAGTCG
     1021 CTGCGACGCT GCCTTCGCCC CGTGCCCCGC TCCGCCGCCG CCTCGCGCCG CCCGCCCCGG
     1081 CTCTGACTGA CCGCGTTACT CCCACAGGTG AGCGGGCGGG ACGGCCCTTC TCCTCCGGGC
     1141 TGTAATTAGC TGAGCAAGAG GTAAGGGTTT AAGGGATGGT TGGTTGGTGG GGTATTAATG
     1201 TTTAATTACC TGGAGCACCT GCCTGAAATC ACTTTTTTTC AGGTTGGACC GGTGCCACCA
     1261 TGGACTATAA GGACCACGAC GGAGACTACA AGGATCATGA TATTGATTAC AAAGACGATG
     1321 ACGATAAGAT GGCCCCAAAG AAGAAGCGGA AGGTCGGTAT CCACGGAGTC CCAGCAGCCG
     1381 ACAAGAAGTA CAGCATCGGC CTGGACATCG GCACCAACTC TGTGGGCTGG GCCGTGATCA
     1441 CCGACGAGTA CAAGGTGCCC AGCAAGAAAT TCAAGGTGCT GGGCAACACC GACCGGCACA
     1501 GCATCAAGAA GAACCTGATC GGAGCCCTGC TGTTCGACAG CGGCGAAACA GCCGAGGCCA
     1561 CCCGGCTGAA GAGAACCGCC AGAAGAAGAT ACACCAGACG GAAGAACCGG ATCTGCTATC
     1621 TGCAAGAGAT CTTCAGCAAC GAGATGGCCA AGGTGGACGA CAGCTTCTTC CACAGACTGG
     1681 AAGAGTCCTT CCTGGTGGAA GAGGATAAGA AGCACGAGCG GCACCCCATC TTCGGCAACA
     1741 TCGTGGACGA GGTGGCCTAC CACGAGAAGT ACCCCACCAT CTACCACCTG AGAAAGAAAC
     1801 TGGTGGACAG CACCGACAAG GCCGACCTGC GGCTGATCTA TCTGGCCCTG GCCCACATGA
     1861 TCAAGTTCCG GGGCCACTTC CTGATCGAGG GCGACCTGAA CCCCGACAAC AGCGACGTGG
     1921 ACAAGCTGTT CATCCAGCTG GTGCAGACCT ACAACCAGCT GTTCGAGGAA AACCCCATCA
     1981 ACGCCAGCGG CGTGGACGCC AAGGCCATCC TGTCTGCCAG ACTGAGCAAG AGCAGACGGC
     2041 TGGAAAATCT GATCGCCCAG CTGCCCGGCG AGAAGAAGAA TGGCCTGTTC GGAAACCTGA
     2101 TTGCCCTGAG CCTGGGCCTG ACCCCCAACT TCAAGAGCAA CTTCGACCTG GCCGAGGATG
     2161 CCAAACTGCA GCTGAGCAAG GACACCTACG ACGACGACCT GGACAACCTG CTGGCCCAGA
     2221 TCGGCGACCA GTACGCCGAC CTGTTTCTGG CCGCCAAGAA CCTGTCCGAC GCCATCCTGC
     2281 TGAGCGACAT CCTGAGAGTG AACACCGAGA TCACCAAGGC CCCCCTGAGC GCCTCTATGA
     2341 TCAAGAGATA CGACGAGCAC CACCAGGACC TGACCCTGCT GAAAGCTCTC GTGCGGCAGC
     2401 AGCTGCCTGA GAAGTACAAA GAGATTTTCT TCGACCAGAG CAAGAACGGC TACGCCGGCT
     2461 ACATTGACGG CGGAGCCAGC CAGGAAGAGT TCTACAAGTT CATCAAGCCC ATCCTGGAAA
     2521 AGATGGACGG CACCGAGGAA CTGCTCGTGA AGCTGAACAG AGAGGACCTG CTGCGGAAGC
     2581 AGCGGACCTT CGACAACGGC AGCATCCCCC ACCAGATCCA CCTGGGAGAG CTGCACGCCA
     2641 TTCTGCGGCG GCAGGAAGAT TTTTACCCAT TCCTGAAGGA CAACCGGGAA AAGATCGAGA
     2701 AGATCCTGAC CTTCCGCATC CCCTACTACG TGGGCCCTCT GGCCAGGGGA AACAGCAGAT
     2761 TCGCCTGGAT GACCAGAAAG AGCGAGGAAA CCATCACCCC CTGGAACTTC GAGGAAGTGG
     2821 TGGACAAGGG CGCTTCCGCC CAGAGCTTCA TCGAGCGGAT GACCAACTTC GATAAGAACC
     2881 TGCCCAACGA GAAGGTGCTG CCCAAGCACA GCCTGCTGTA CGAGTACTTC ACCGTGTATA
     2941 ACGAGCTGAC CAAAGTGAAA TACGTGACCG AGGGAATGAG AAAGCCCGCC TTCCTGAGCG
     3001 GCGAGCAGAA AAAGGCCATC GTGGACCTGC TGTTCAAGAC CAACCGGAAA GTGACCGTGA
     3061 AGCAGCTGAA AGAGGACTAC TTCAAGAAAA TCGAGTGCTT CGACTCCGTG GAAATCTCCG
     3121 GCGTGGAAGA TCGGTTCAAC GCCTCCCTGG GCACATACCA CGATCTGCTG AAAATTATCA
     3181 AGGACAAGGA CTTCCTGGAC AATGAGGAAA ACGAGGACAT TCTGGAAGAT ATCGTGCTGA
     3241 CCCTGACACT GTTTGAGGAC AGAGAGATGA TCGAGGAACG GCTGAAAACC TATGCCCACC
     3301 TGTTCGACGA CAAAGTGATG AAGCAGCTGA AGCGGCGGAG ATACACCGGC TGGGGCAGGC
     3361 TGAGCCGGAA GCTGATCAAC GGCATCCGGG ACAAGCAGTC CGGCAAGACA ATCCTGGATT
     3421 TCCTGAAGTC CGACGGCTTC GCCAACAGAA ACTTCATGCA GCTGATCCAC GACGACAGCC
     3481 TGACCTTTAA AGAGGACATC CAGAAAGCCC AGGTGTCCGG CCAGGGCGAT AGCCTGCACG
     3541 AGCACATTGC CAATCTGGCC GGCAGCCCCG CCATTAAGAA GGGCATCCTG CAGACAGTGA
     3601 AGGTGGTGGA CGAGCTCGTG AAAGTGATGG GCCGGCACAA GCCCGAGAAC ATCGTGATCG
     3661 AAATGGCCAG AGAGAACCAG ACCACCCAGA AGGGACAGAA GAACAGCCGC GAGAGAATGA
     3721 AGCGGATCGA AGAGGGCATC AAAGAGCTGG GCAGCCAGAT CCTGAAAGAA CACCCCGTGG
```

3781 AAAACACCCA GCTGCAGAAC GAGAAGCTGT ACCTGTACTA CCTGCAGAAT GGGCGGGATA
3841 TGTACGTGGA CCAGGAACTG GACATCAACC GGCTGTCCGA CTACGATGTG GACCATATCG
3901 TGCCTCAGAG CTTTCTGAAG GACGACTCCA TCGACAACAA GGTGCTGACC AGAAGCGACA
3961 AGAACCGGGG CAAGAGCGAC AACGTGCCCT CCGAAGAGGT CGTGAAGAAG ATGAAGAACT
4021 ACTGGCGGCA GCTGCTGAAC GCCAAGCTGA TTACCCAGAG AAAGTTCGAC AATCTGACCA
4081 AGGCCGAGAG AGGCGGCCTG AGCGAACTGG ATAAGGCCGG CTTCATCAAG AGACAGCTGG
4141 TGGAAACCCG GCAGATCACA AAGCACGTGG CACAGATCCT GGACTCCCGG ATGAACACTA
4201 AGTACGACGA GAATGACAAG CTGATCCGGG AAGTGAAAGT GATCACCCTG AAGTCCAAGC
4261 TGGTGTCCGA TTTCCGGAAG GATTTCCAGT TTTACAAAGT GCGCGAGATC AACAACTACC
4321 ACCACGCCCA CGACGCCTAC CTGAACGCCG TCGTGGGAAC CGCCCTGATC AAAAAGTACC
4381 CTAAGCTGGA AAGCGAGTTC GTGTACGGCG ACTACAAGGT GTACGACGTG CGGAAGATGA
4441 TCGCCAAGAG CGAGCAGGAA ATCGGCAAGG CTACCGCCAA GTACTTCTTC TACAGCAACA
4501 TCATGAACTT TTTCAAGACC GAGATTACCC TGGCCAACGG CGAGATCCGG AAGCGGCCTC
4561 TGATCGAGAC AAACGGCGAA ACCGGGGAGA TCGTGTGGGA TAAGGGCCGG GATTTTGCCA
4621 CCGTGCGGAA AGTGCTGAGC ATGCCCCAAG TGAATATCGT GAAAAAGACC GAGGTGCAGA
4681 CAGGCGGCTT CAGCAAAGAG TCTATCCTGC CCAAGAGGAA CAGCGATAAG CTGATCGCCA
4741 GAAAGAAGGA CTGGGACCCT AAGAAGTACG GCGGCTTCGA CAGCCCCACC GTGGCCTATT
4801 CTGTGCTGGT GGTGGCCAAA GTGGAAAAGG GCAAGTCCAA GAAACTGAAG AGTGTGAAAG
4861 AGCTGCTGGG GATCACCATC ATGGAAAGAA GCAGCTTCGA GAAGAATCCC ATCGACTTTC
4921 TGGAAGCCAA GGGCTACAAA GAAGTGAAAA AGGACCTGAT CATCAAGCTG CCTAAGTACT
4981 CCCTGTTCGA GCTGGAAAAC GGCCGGAAGA GAATGCTGGC CTCTGCCGGC GAACTGCAGA
5041 AGGGAAACGA ACTGGCCCTG CCCTCCAAAT ATGTGAACTT CCTGTACCTG GCCAGCCACT
5101 ATGAGAAGCT GAAGGGCTCC CCCGAGGATA ATGAGCAGAA ACAGCTGTTT GTGGAACAGC
5161 ACAAGCACTA CCTGGACGAG ATCATCGAGC AGATCAGCGA GTTCTCCAAG AGAGTGATCC
5221 TGGCCGACGC TAATCTGGAC AAAGTGCTGT CCGCCTACAA CAAGCACCGG GATAAGCCCA
5281 TCAGAGAGCA GGCCGAGAAT ATCATCCACC TGTTTACCCT GACCAATCTG GGAGCCCCTG
5341 CCGCCTTCAA GTACTTTGAC ACCACCATCG ACCGGAAGAG GTACACCAGC ACCAAAGAGG
5401 TGCTGGACGC CACCCTGATC CACCAGAGCA TCACCGGCCT GTACGAGACA CGGATCGACC
5461 TGTCTCAGCT GGGAGGCGAC AAAAGGCCGG CGGCCACGAA AAAGGCCGGC CAGGCAAAAA
5521 AGAAAAAGCT TGAGGGCAGA GGAAGTCTGC TAACATGCGG TGACGTGGAG GAGAATCCCG
5581 GCCCTGCTAG CATGGTGAGC AAGGGCGAGG AGCTGTTCAC CGGGGTGGTG CCCATCCTGG
5641 TCGAGCTGGA CGGCGACGTA AACGGCCACA AGTTCAGCGT GAGGGGCGAG GGCGAGGGCG
5701 ATGCCACCAA CGGCAAGCTG ACCCTGAAGT TCATCTGCAC CACCGGCAAG CTGCCCGTGC
5761 CCTGGCCCAC CCTCGTGACC ACCCTGAGCC ACGGCGTGCA GTGCTTCGCC CGCTACCCCG
5821 ACCACATGAA GCAGCACGAC TTCTTCAAGT CCGCCATGCC CGAAGGCTAC GTCCAGGAGC
5881 GCACCATCTT CTTCAAGGAC GACGGCACCT ACAAGACCCG CGCCGAGGTG AAGTTCGAGG
5941 GCGACACCCT GGTGAACCGC ATCGAGCTGA AGGGCGTCGA CTTCAAGGAG GACGGCAACA
6001 TCCTGGGGCA CAAGCTGGAG TACAACTTCA ACAGCCACAA CATCTATATC ATGGCCGTCA
6061 AGCAGAAGAA CGGCATCAAG GTGAACTTCA AGATCCGCCA CAACGTGGAG GACGGCAGCG
6121 TGCAGCTCGC CGACCACTAC CAGCAGAACA CCCCCATCGG CGACGGCCCC GTGCTGCTGC
6181 CCGACAGCCA CTACCTGAGC ACCCAGTCCG TGCTGAGCAA AGACCCCAAC GAGAAGCGCG
6241 ATCACATGGT CCTGCTGGAG TTCCGCACCG CCGCCGGGAT CACTCTCGGC ATGGACGAGC
6301 TGTACAAGTG AGAATTCCTA GAGCTCGCTG ATCAGCCTCG ACTGTGCCTT CTAGTTGCCA
6361 GCCATCTGTT GTTTGCCCCT CCCCCGTGCC TTCCTTGACC CTGGAAGGTG CCACTCCCAC
6421 TGTCCTTTCC TAATAAAATG AGGAAATTGC ATCGCATTGT CTGAGTAGGT GTCATTCTAT
6481 TCTGGGGGGT GGGGTGGGGC AGGACAGCAA GGGGGAGGAT TGGGAAGAGA ATAGCAGGCA
6541 TGCTGGGGAG CGGCCGCAGG AACCCCTAGT GATGGAGTTG GCCACTCCCT CTCTGCGCGC
6601 TCGCTCGCTC ACTGAGGCCG GGCGACCAAA GGTCGCCCGA CGCCCGGGCT TTGCCCGGGC
6661 GGCCTCAGTG AGCGAGCGAG CGCGCAGCTG CCTGCAGGGG CGCCTGATGC GGTATTTTCT
6721 CCTTACGCAT CTGTGCGGTA TTTCACACCG CATACGTCAA AGCAACCATA GTACGCGCCC
6781 TGTAGCGGCG CATTAAGCGC GGCGGGTGTG GTGGTTACGC GCAGCGTGAC CGCTACACTT
6841 GCCAGCGCCC TAGCGCCCGC TCCTTTCGCT TTCTTCCCTT CCTTTCTCGC CACGTTCGCC
6901 GGCTTTCCCC GTCAAGCTCT AAATCGGGGG CTCCCTTTAG GGTTCCGATT TAGTGCTTTA
6961 CGGCACCTCG ACCCCAAAAA ACTTGATTTG GGTGATGGTT CACGTAGTGG GCCATCGCCC
7021 TGATAGACGG TTTTTCGCCC TTTGACGTTG GAGTCCACGT TCTTTAATAG TGGACTCTTG
7081 TTCCAAACTG GAACAACACT CAACCCTATC TCGGGCTATT CTTTTGATTT ATAAGGGATT
7141 TTGCCGATTT CGGCCTATTG GTTAAAAAAT GAGCTGATTT AACAAAAATT TAACGCGAAT
7201 TTTAACAAAA TATTAACGTT TACAATTTTA TGGTGCACTC TCAGTACAAT CTGCTCTGAT
7261 GCCGCATAGT TAAGCCAGCC CCGACACCCG CCAACACCCG CTGACGCGCC CTGACGGGCT
7321 TGTCTGCTCC CGGCATCCGC TTACAGACAA GCTGTGACCG TCTCCGGGAG CTGCATGTGT
7381 CAGAGGTTTT CACCGTCATC ACCGAAACGC GCGAGACGAA AGGGCCTCGT GATACGCCTA
7441 TTTTTATAGG TTAATGTCAT GATAATAATG GTTTCTTAGA CGTCAGGTGG CACTTTTCGG
7501 GGAAATGTGC GCGGAACCCC TATTTGTTTA TTTTTCTAAA TACATTCAAA TATGTATCCG
7561 CTCATGAGAC AATAACCCTG ATAAATGCTT CAATAATATT GAAAAAGGAA GAGTATGAGT
7621 ATTCAACATT TCCGTGTCGC CCTTATTCCC TTTTTTGCGG CATTTTGCCT TCCTGTTTTT
7681 GCTCACCCAG AAACGCTGGT GAAAGTAAAA GATGCTGAAG ATCAGTTGGG TGCACGAGTG
7741 GGTTACATCG AACTGGATCT CAACAGCGGT AAGATCCTTG AGAGTTTTCG CCCCGAAGAA
7801 CGTTTTCCAA TGATGAGCAC TTTTAAAGTT CTGCTATGTG GCGCGGTATT ATCCCGTATT
7861 GACGCCGGGC AAGAGCAACT CGGTCGCCGC ATACACTATT CTCAGAATGA CTTGGTTGAG
7921 TACTCACCAG TCACAGAAAA GCATCTTACG GATGGCATGA CAGTAAGAGA ATTATGCAGT

7981 GCTGCCATAA CCATGAGTGA TAACACTGCG GCCAACTTAC TTCTGACAAC GATCGGAGGA
8041 CCGAAGGAGC TAACCGCTTT TTTGCACAAC ATGGGGGATC ATGTAACTCG CCTTGATCGT
8101 TGGGAACCGG AGCTGAATGA AGCCATACCA AACGACGAGC GTGACACCAC GATGCCTGTA
8161 GCAATGGCAA CAACGTTGCG CAAACTATTA ACTGGCGAAC TACTTACTCT AGCTTCCCGG
8221 CAACAATTAA TAGACTGGAT GGAGGCGGAT AAAGTTGCAG GACCACTTCT GCGCTCGGCC
8281 CTTCCGGCTG GCTGGTTTAT TGCTGATAAA TCTGGAGCCG GTGAGCGTGG AAGCCGCGGT
8341 ATCATTGCAG CACTGGGGCC AGATGGTAAG CCCTCCCGTA TCGTAGTTAT CTACACGACG
8401 GGGAGTCAGG CAACTATGGA TGAACGAAAT AGACAGATCG CTGAGATAGG TGCCTCACTG
8461 ATTAAGCATT GGTAACTGTC AGACCAAGTT TACTCATATA TACTTTAGAT TGATTTAAAA
8521 CTTCATTTTT AATTTAAAAG GATCTAGGTG AAGATCCTTT TTGATAATCT CATGACCAAA
8581 ATCCCTTAAC GTGAGTTTTC GTTCCACTGA GCGTCAGACC CCGTAGAAAA GATCAAAGGA
8641 TCTTCTTGAG ATCCTTTTTT TCTGCGCGTA ATCTGCTGCT TGCAAACAAA AAAACCACCG
8701 CTACCAGCGG TGGTTTGTTT GCCGGATCAA GAGCTACCAA CTCTTTTTCC GAAGGTAACT
8761 GGCTTCAGCA GAGCGCAGAT ACCAAATACT GTCCTTCTAG TGTAGCCGTA GTTAGGCCAC
8821 CACTTCAAGA ACTCTGTAGC ACCGCCTACA TACCTCGCTC TGCTAATCCT GTTACCAGTG
8881 GCTGCTGCCA GTGGCGATAA GTCGTGTCTT ACCGGGTTGG ACTCAAGACG ATAGTTACCG
8941 GATAAGGCGC AGCGGTCGGG CTGAACGGGG GGTTCGTGCA CACAGCCCAG CTTGGAGCGA
9001 ACGACCTACA CCGAACTGAG ATACCTACAG CGTGAGCTAT GAGAAAGCGC CACGCTTCCC
9061 GAAGGGAGAA AGGCGGACAG GTATCCGGTA AGCGGCAGGG TCGGAACAGG AGAGCGCACG
9121 AGGGAGCTTC CAGGGGGAAA CGCCTGGTAT CTTTATAGTC CTGTCGGGTT TCGCCACCTC
9181 TGACTTGAGC GTCGATTTTT GTGATGCTCG TCAGGGGGGC GGAGCCTATG GAAAAACGCC
9241 AGCAACGCGG CCTTTTTACG GTTCCTGGCC TTTTGCTGGC CTTTTGCTCA CATGT
